# Heterotrimeric Go protein links Wnt-Frizzled signaling with ankyrins to regulate the neuronal microtubule cytoskeleton

**DOI:** 10.1242/dev.106773

**Published:** 2014-09

**Authors:** Anne-Marie Lüchtenborg, Gonzalo P. Solis, Diane Egger-Adam, Alexey Koval, Chen Lin, Maxime G. Blanchard, Stephan Kellenberger, Vladimir L. Katanaev

**Affiliations:** 1 Department of Pharmacology and Toxicology, Faculty of Biology and Medicine, University of Lausanne, Rue du Bugnon 27, Lausanne 1005, Switzerland; 2 Department of Biology, University of Konstanz, Universitätsstrasse 10, Box 643, Konstanz 78457, Germany

**Keywords:** *Drosophila*, Neuromuscular junction, Wnt, Frizzled, G protein, Ankyrin, Microtubules

## Abstract

*Drosophila* neuromuscular junctions (NMJs) represent a powerful model system with which to study glutamatergic synapse formation and remodeling. Several proteins have been implicated in these processes, including components of canonical Wingless (*Drosophila* Wnt1) signaling and the giant isoforms of the membrane-cytoskeleton linker Ankyrin 2, but possible interconnections and cooperation between these proteins were unknown. Here, we demonstrate that the heterotrimeric G protein Go functions as a transducer of Wingless-Frizzled 2 signaling in the synapse. We identify Ankyrin 2 as a target of Go signaling required for NMJ formation. Moreover, the Go-ankyrin interaction is conserved in the mammalian neurite outgrowth pathway. Without ankyrins, a major switch in the Go-induced neuronal cytoskeleton program is observed, from microtubule-dependent neurite outgrowth to actin-dependent lamellopodial induction. These findings describe a novel mechanism regulating the microtubule cytoskeleton in the nervous system. Our work in *Drosophila* and mammalian cells suggests that this mechanism might be generally applicable in nervous system development and function.

## INTRODUCTION

Go is the most abundant heterotrimeric G protein in the central nervous system of both vertebrates and invertebrates (Sternweis and Robishaw, 1984; Wolfgang et al., 1990). It is the immediate transducer of a number of G protein-coupled receptors (GPCRs), including receptors of the Frizzled (Fz) family (Egger-Adam and Katanaev, 2008). In *Drosophila*, Go is involved in transduction of the Wingless (Wg; *Drosophila* Wnt1) signal (Katanaev et al., 2005). Go can physically interact with Fz proteins, and binding of Wnt ligands to Fz induces an exchange of the guanine nucleotide on the Gα subunit of Go (Gαo) (Koval and Katanaev, 2011). The initial heterotrimeric complex then dissociates into free Gα-GTP and the Gβγ dimer; both are involved in downstream signaling. The intrinsic GTPase activity of Gα leads to hydrolysis of GTP to GDP; the resultant Gα-GDP can continue to signal or associates back with Gβγ to bind GPCRs (Gilman, 1987; Katanaev, 2010).

The evolutionarily conserved Wg pathway is important for numerous developmental programs and cellular processes (Logan and Nusse, 2004). In the nervous system of *Drosophila*, Wg signaling is involved in the formation of neuromuscular junctions (NMJs) (Packard et al., 2002; Miech et al., 2008). Being a glutamatergic synapse, the *Drosophila* NMJ provides a useful experimental model with which to study mammalian central nervous system synapses, their formation and remodeling (Collins and DiAntonio, 2007). The *Drosophila* NMJ is a beads-on-a-string-like structure that is formed at the axon terminus and is composed of distinct circular structures – the synaptic boutons – which contain active zones for neurotransmitter release. During growth, the NMJ is subject to remodeling to build additional synapses on the growing muscle, which is achieved by the formation of new boutons as well as by budding off from the existing boutons (Zito et al., 1999). These processes require cytoskeletal rearrangements (Roos et al., 2000) and depend on the proper response to the Wg ligand, which is produced presynaptically (Packard et al., 2002; Korkut et al., 2009).

In canonical Wnt signaling, binding of the ligand to Fz and a co-receptor, LRP5/6 (Arrow in *Drosophila*), leads to reorganization of the cytoplasmic β-catenin-destruction machinery, which contains, among other proteins, glycogen synthase kinase 3β [GSK3β; Shaggy (Sgg) in *Drosophila*]. Receptors (Fz and LRP5/6) are activated by Wnt signal to disassemble the destruction complex, leading to the stabilization of β-catenin, its translocation into the nucleus and the induction of transcription of Wnt target genes (Logan and Nusse, 2004).

However, this canonical pathway is not active in the *Drosophila* NMJ. Instead, on the postsynaptic side of the NMJ the Wg signal is transduced via endocytosis and cleavage of Frizzled 2 (Fz2) and nuclear import of its C-terminal fragment, which is required for the proper transcription-dependent establishment of postsynaptic densities (Mathew et al., 2005; Mosca and Schwarz, 2010). On the presynaptic side, the Wg pathway does not involve β-catenin nor transcription but does require inhibition of Sgg activity (Miech et al., 2008); Sgg in the presynapse is proposed to regulate the stability of the microtubule cytoskeleton through phosphorylation of the microtubule-binding protein Futsch (*Drosophila* MAP1B) (Franco et al., 2004; Gogel et al., 2006; Miech et al., 2008). The microtubule cytoskeleton in the presynaptic NMJ cell is also under the control of Ankyrin 2 (Hortsch et al., 2002; Koch et al., 2008; Pielage et al., 2008).

Ankyrins (Ank) are highly abundant modular proteins that mediate protein-protein interactions, mainly serving as adaptors for linking the cytoskeleton to the plasma membrane (Bennett and Baines, 2001). Mammalian genomes encode three Ank genes [*AnkR* (*Ank1*), *AnkB* (*Ank2*) and *AnkG* (*Ank3*)], whereas *Drosophila* has two [*Ank1* (also known as *Ank* – FlyBase) and *Ank2*] (Dubreuil and Yu, 1994; Bouley et al., 2000). *Ank2* is expressed exclusively in neurons and exists in several splicing variants (Koch et al., 2008; Pielage et al., 2008). The larger isoforms (Ank2M, Ank2L and Ank2XL) are localized to axons and play important roles in NMJ formation and function (Hortsch et al., 2002; Koch et al., 2008; Pielage et al., 2008). The C-terminal part of Ank2L can bind to microtubules (Pielage et al., 2008). Despite the well-established role of Ank2 in NMJ formation, its function has been considered somewhat passive and its mode of regulation has not been clarified. Here, we show that Gαo binds to Ank2 and that these proteins and the Wg pathway components Wg, Fz2, and Sgg jointly coordinate the formation of the NMJ. We also show that the functional Gαo-Ank interaction is conserved from insects to mammals.

## RESULTS

### Go is abundant in the NMJ and is required for normal NMJ physiology

**Fig. 1.**
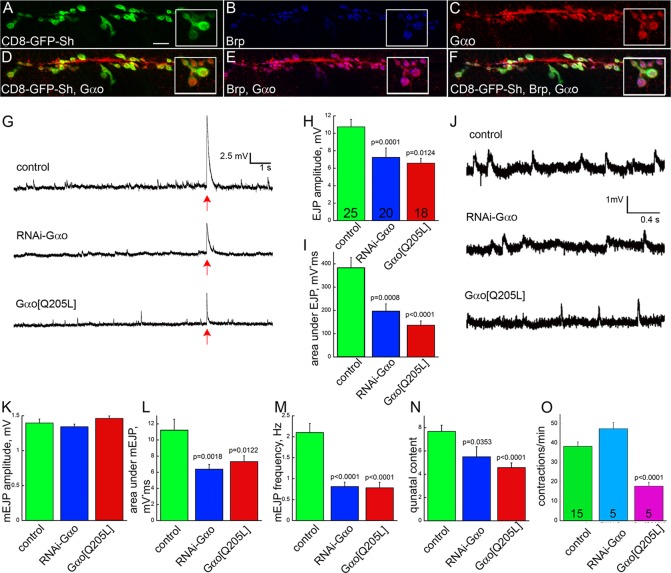
**Gαo is expressed in the presynaptic cell of the NMJ and is required for normal NMJ physiology.** (A-F) Gαo (red in C-F) is expressed in the presynaptic side of the NMJ and is barely detected postsynaptically, as judged by colocalization with Brp (blue in B,E,F) but only partial overlap with CD8-GFP-Sh (green in A,D,F). Insets are enlargements of the terminal boutons. Scale bar: 10 μm. (G) Representative traces of spontaneous NMJ activity and one illumination-evoked action potential [arrow indicates the time of illumination; arrow thickness is in scale with the length of illumination (20 ms)] recorded from control (*OK371-Gal4;UAS-ChR2*), RNAi-Gαo (*OK371-Gal4;UAS-ChR2/UAS-RNAi-Gαo*) and Gαo[Q205L] (*OK371-Gal4;UAS-ChR2/UAS-Gαo[Q205L]*) larvae. (H,I) Quantification of amplitude (H) and area under the peak (I) of excitatory junctional potentials (EJPs) from individual muscles from the three genotypes; the number of muscles analyzed is shown in H. (J) Higher magnification of a region in G to show representative traces of spontaneous NMJ activity. (K-M) Quantification of amplitude (K), area (L) and frequency (M) of spontaneous miniature EJPs (mEJPs) of the three genotypes, recorded in the same muscles as in H. (N) Quantal content of the three genotypes calculated as EJP/mEJP. (O) Locomotion activity measured as the number of contractions per minute of third instar larvae of the three genotypes; the number of animals tested is shown in the bars. *P*-values are shown where the observed differences between the mutant and control conditions are statistically significant (*P*<0.05). Error bars indicate s.e.m.

Since Go is abundant in neurons and is involved in Fz signaling, we investigated its presence and function in the NMJ. To visualize the synaptic boutons, we used the postsynaptic marker CD8-GFP-Sh (Zito et al., 1999) or Discs large (Dlg; Dlg1 – FlyBase) (Guan et al., 1996) (Fig. 1A; supplementary material Fig. S1A). For the presynaptic side we used the marker Bruchpilot (Brp) (Wagh et al., 2006) (Fig. 1B) or performed anti-HRP staining (Jan and Jan, 1982) (supplementary material Fig. S1A,B). Using two different anti-Gαo antibodies (see Materials and Methods), we found strong anti-Gαo staining in boutons as well as in axons (Fig. 1C; supplementary material Fig. S1A-D). Comparison of Gαo staining with the markers revealed that Gαo is expressed in the presynaptic cell, overlapping with Brp (Fig. 1D-F; supplementary material Fig. S1C,D) and anti-HRP (supplementary material Fig. S1A,B). This is particularly evident at high magnification, which shows the anti-Gαo staining encircled by postsynaptic Dlg and CD8-GFP-Sh (supplementary material Fig. S1B,D). Interestingly, this pattern is different from that of anti-Gβ13F staining, which recognizes the major Gβ subunit in *Drosophila* (Katanayeva et al., 2010): this pan-G protein Gβ subunit shows both pre- and postsynaptic staining, the latter being even broader than the CD8-GFP-Sh pattern (supplementary material Fig. S1H) or that of anti-Dlg (not shown). A role of Gβ13F both in the nervous system (Schaefer et al., 2001) and in muscles (Schnorrer et al., 2010) has been described previously.

To investigate the physiological importance of Gαo in the NMJ, we perturbed Gαo activity in the synapse. Gαo was modulated by the presynaptic expression of two previously tested *UAS* constructs: *RNAi-Gαo*, which downregulates Gαo (Purvanov et al., 2010) (see supplementary material Fig. S1I-K for the efficiency of downregulation); and *Gαo[Q205L]*, which is a constitutively active mutant form that is unable to hydrolyze GTP (Katanaev et al., 2005; Kopein and Katanaev, 2009). These two constructs were driven by the motoneuron driver *OK371-Gal4* (Mahr and Aberle, 2006). Excitatory junctional potentials (EJPs) were induced by light-activated channelrhodopsin-2 (Schroll et al., 2006) (see Materials and Methods). Analysis of EJPs in the NMJ of the control, *RNAi-Gαo* and *Gαo[Q205L]* larvae revealed a marked reduction in EJP amplitude and width with each perturbation of Gαo function (Fig. 1G-I).

We also analyzed spontaneous NMJ activity. Although the amplitude of miniature excitatory junctional potentials (mEJPs) was almost identical in the three conditions, their duration and frequency were strongly reduced upon overactivation and downregulation of Gαo (Fig. 1J-M). Decreased mEJP frequency with largely unperturbed mEJP amplitude suggests that motoneuron-specific modulation of Gαo function mainly induces presynaptic defects. The ratio of EJP to mEJP amplitudes provides the junctional quantal content. This measure of synaptic efficacy is significantly reduced in both mutant conditions (Fig. 1N), suggesting that the number of synaptic vesicles released upon stimulation is decreased in the *RNAi-Gαo* and *Gαo[Q205L]* conditions. These data might indicate that the number of mature boutons or their functionality is decreased by unbalancing Gαo activity in the presynapse. Additionally, we found that in *Gαo[Q205L]* larvae the overall crawling capacity was also perturbed (Fig. 1O).

### Aberrant Gαo activity leads to morphological defects in the NMJ similar to those associated with abnormal Wg-Fz2 signaling

**Fig. 2.**
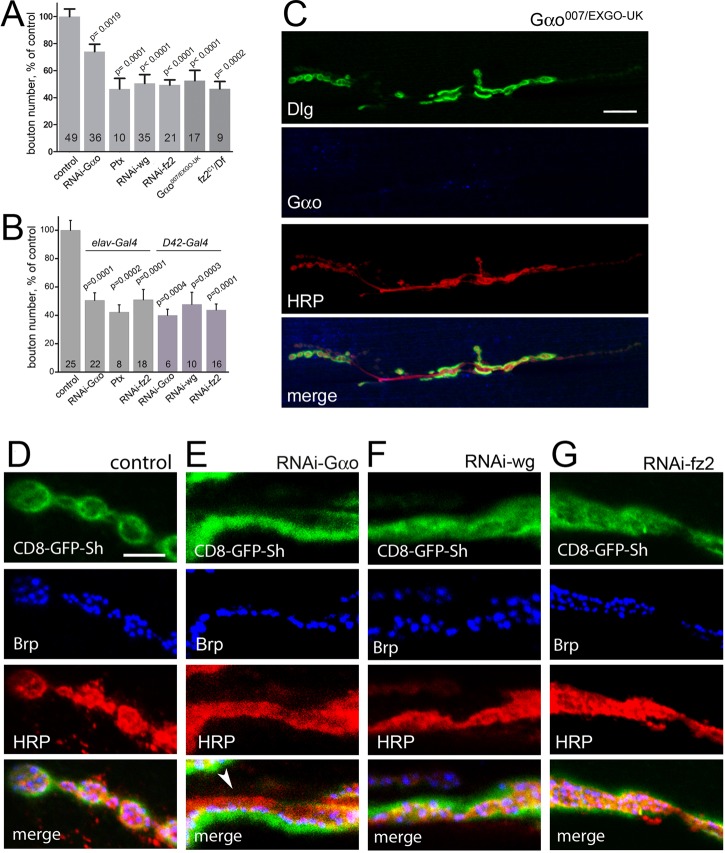
**Gαo is required for NMJ formation, similar to Wg and Fz2.** (A) Quantitative analysis of bouton number on muscle 6/7. Presynaptic downregulation of Gαo, Wg and Fz2 with the driver *OK371-Gal4*, expression of Ptx, as well as genetic removal of *Gαo* or *Fz2* lead to a significant decrease in bouton number compared with the wild type (control). Data are represented as percentage of control; the number of NMJs analyzed for each genotype is shown in each bar; *P*-values compared with the control are indicated; error bars indicate s.e.m. (B) Downregulation of Gαo or Fz2 and expression of Ptx with the pan-neuronal driver *elav-Gal4* similarly decrease bouton numbers. The same effect is observed when RNAi against *Gαo*, *wg* or *fz2* is driven with motoneuron-specific *D42-Gal4*. (C) Genetic removal of *Gαo* leads to a strong reduction in bouton number and aberrant NMJ morphology (compare with Fig. 1A-F). Anti-Gαo staining (blue) confirms loss of the proteins; the remaining signal is non-specific. (D-G) Presynaptic downregulation of Gαo, Wg or Fz2 results in malformed boutons. Displayed is a detail of the NMJ on muscle 6/7 that is stained with anti-HRP to visualize the presynaptic cell membrane in red, with anti-Brp to stain the active zones in blue, and the postsynaptic marker CD8-GFP-Sh in green. All mutant genotypes lead to the development of elongated structures with defective overlap of pre- and postsynapse (E, arrowhead) instead of the circular postsynaptic boutons with postsynaptic staining encircling presynaptic staining as in the wild type (D). Scale bars: 20 µm in C; 5 µm in D-G.

To examine why aberrant NMJ physiology accompanies reduced or increased Gαo activity, we performed immunostaining and a morphological investigation of the mutant synapses. We found reduced numbers of boutons in *RNAi-Gαo*-expressing NMJs (Fig. 2A). This reduction was rescued by re-expression of Gαo (but not of an unrelated protein; supplementary material Fig. S2A). Pertussis toxin (Ptx) is a specific inhibitor of Gαo in *Drosophila*, uncoupling it from cognate GPCRs (Katanaev and Tomlinson, 2006b), and its expression in motoneurons led to a ∼50% reduction in the number of boutons (Fig. 2A). In addition to *OK371-Gal4*, other drivers such as the pan-neuronal *elav-Gal4* (Luo et al., 1994) (see supplementary material Fig. S1K,L) and the motoneuron-specific *D42-Gal4* (Parkes et al., 1998), when used to target Gαo through expression of RNAi or Ptx, also led to a substantial decrease in bouton numbers (Fig. 2B). The Wg-secreting type Ib boutons (Packard et al., 2002) appeared more severely affected by Gαo perturbations than type Is boutons (supplementary material Fig. S2B). Finally, genetic removal of *Gαo* replicated the Gαo downregulation data (Fig. 2A), resulting in a strong reduction in bouton numbers and aberrant NMJ morphology (Fig. 2C, compare with Fig. 1A-F); presynaptic re-expression of Gαo was able to rescue the *Gαo^−/−^* defects (supplementary material Fig. S2A). Thus, Gαo is presynaptically required for proper NMJ development. The decrease in bouton number induced by *RNAi-Gαo* parallels the reduced electric activity of the mutant NMJ (Fig. 1).

Gαo is a transducer of Fz2 (Katanaev et al., 2005; Katanaev and Tomlinson, 2006a; Purvanov et al., 2010), and the Wg-Fz2 pathway has been implicated in NMJ formation. In accordance with previous observations (Packard et al., 2002; Mathew et al., 2005), presynaptic downregulation of Wg (supplementary material Fig. S1M,N) or genetic loss of *fz2* led to a strong decrease in bouton numbers (Fig. 2A,B). Fz2 is present both pre- and postsynaptically (Packard et al., 2002), and the importance of the postsynaptic Fz2 for NMJ development has been demonstrated (Mathew et al., 2005; Mosca and Schwarz, 2010). Here we show that presynaptic Fz2 is also crucial for the NMJ, as specific presynaptic downregulation of Fz2 by various drivers (supplementary material Fig. S1O,P) reduces bouton numbers to the levels found in *fz2* null mutants (Fig. 2A,B). We also tested the ability of presynaptic re-expression of *fz2* to rescue bouton numbers in the *fz2* null background, and observed a complete rescue of bouton number (supplementary material Fig. S1Q,R), analogous to the rescue by postsynaptic *fz2* expression in *fz2* mutants (supplementary material Fig. S1Q) (Mathew et al., 2005), providing evidence for the important neuronal role of the Wg-Fz2 pathway in the NMJ.

This quantitative analysis was corroborated with morphological studies. Genetic removal of *Gαo* (Fig. 2C), expression of Ptx (supplementary material Fig. S1S) or silencing of *Gαo* resulted in clear morphological changes in the NMJ (Fig. 2D,E), similar to those previously described for *wg* loss-of-function mutations (Packard et al., 2002) and identical to those induced by downregulation of Wg and Fz2 (Fig. 2F,G), in which tube-like structures could be observed in the mutant NMJs instead of the normal separate circular boutons, often with diffuse presynaptic Brp and anti-HRP staining.

**Fig. 3.**
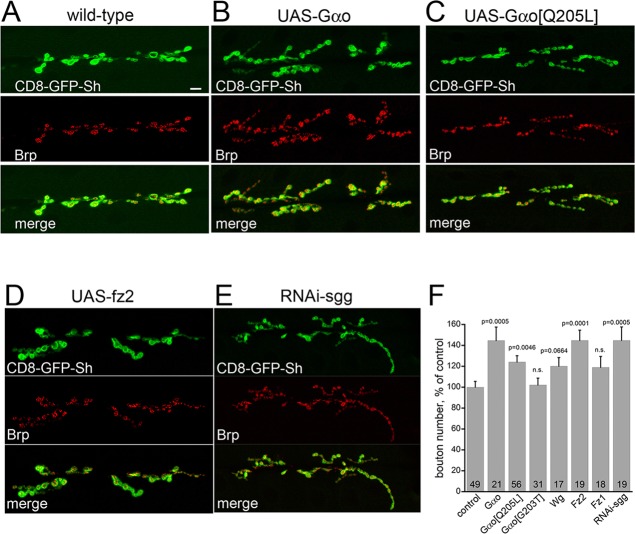
**Overexpression of Gαo or Fz2 in the presynaptic cell, as well as downregulation of Sgg, stimulates bouton formation in NMJ.** (A) Wild-type NMJ stained for the presynaptic marker Brp (red); the postsynaptic cell is visualized by CD8-GFP-Sh (green). (B,C) Overexpression of Gαo and its mutant GTP-loaded form (Gαo[Q205L]) in the presynaptic cell leads to enhanced bouton formation. (D,E) Overexpression of *fz2* or expression of *RNAi-sgg* produces similar phenotypes. (F) Quantification of bouton numbers in the different genotypes (shown as in Fig. 2A). n.s., not significant (*P*>0.05). Scale bar: 10 µm.

We next examined the effect of overexpression of different forms of Gαo in the presynapse. In addition to the constitutively GTP-loaded Gαo[Q205L] form used above, we also overexpressed wild-type Gαo and the Gαo[G203T] mutant (Katanaev et al., 2005), which has a reduced affinity for GTP (supplementary material Fig. S2C) but does not behave as a dominant-negative construct (see Discussion). Expression of all three *Gαo* forms with *OK371-Gal4* induced the formation of smaller and more compact boutons as compared with the normal NMJ (Fig. 3A-C). This morphological change was also observed when *wg* (Packard et al., 2002; Miech et al., 2008) or *fz2* was overexpressed presynaptically (Fig. 3D). Overexpression of *fz1* (also known as *fz* – FlyBase), by contrast, did not affect NMJ morphology (not shown). To further verify the influence of Wg signaling on NMJ formation we expressed *RNAi-sgg* in the presynapse, where Sgg localizes (Franco et al., 2004; Miech et al., 2008). Downregulation of this destruction complex protein resulted in a phenotype similar to that of overexpression of *Gαo* or *fz2* (Fig. 3E).

Quantitative analysis showed that overexpression of *Gαo* and its mutant forms, as well as overexpression of *wg* or *fz2* (but not *fz1*) and downregulation of *sgg*, significantly increased the total number of boutons and their density (the number of boutons per µm NMJ length; Fig. 3F; supplementary material Fig. S2D,E). Expression of different dominant-negative constructs of Sgg (SggDN) presynaptically was previously reported to increase bouton number, whereas postsynaptic expression of SggDN had no effect on NMJ formation (Franco et al., 2004; Miech et al., 2008). As the neurotransmitter release properties of Gαo[Q205L] NMJ are reduced (Fig. 1), the increased numbers of boutons observed upon overactivation of the Wnt pathway, as described here, might indicate that these boutons are non-functional or that Gαo overactivation interferes with proper synaptic transmission.

Cumulatively, these findings suggest that Gαo acts as a transducer of the Wg-Fz2 pathway in the NMJ. Formally, Gαo might alternatively regulate Fz2 abundance in the NMJ. However, no discernible changes in Fz2 levels in the NMJ could be observed in the different *Gαo* backgrounds (supplementary material Fig. S2G).

### Gαo is a transducer of Wg and Fz2 in the NMJ

**Fig. 4.**
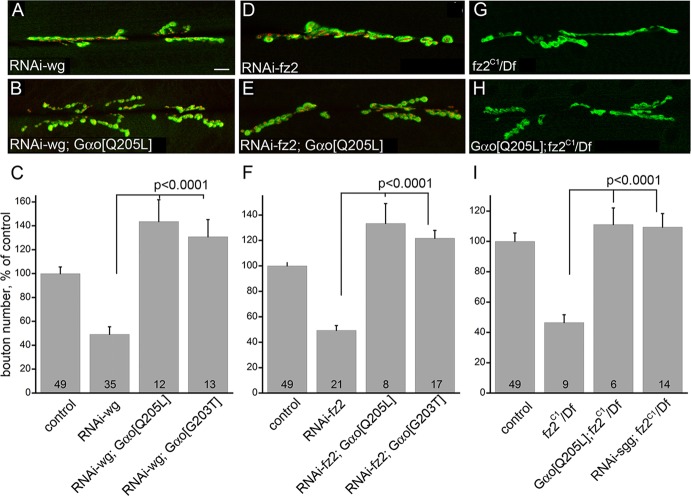
**Gαo acts downstream of Wg-Fz2 in the NMJ.** Expression of the GDP-loaded and GTP-loaded mutant forms of Gαo (Gαo[G203T] and Gαo[Q205L], respectively) rescues the *RNAi-wg* (A-C), *RNAi-fz2* (D-F) and the *fz2* mutant (G-I) phenotypes. Brp (red) and CD8-GFP-Sh (green, A,B,D,E) or Dlg (G,H) visualize pre- and postsynaptic compartments, respectively. Quantification of bouton numbers (C,F,I) is as in Fig. 2A; *RNAi-sgg* also rescues the *fz2* null phenotype (I). Scale bar: 10 µm.

To unequivocally demonstrate that Gαo is a downstream transducer of the Wg-Fz2 signal in the NMJ, we performed epistasis experiments among these proteins. Remarkably, regardless of its nucleotide state, overexpression of *Gαo* in the motoneurons was effective in rescuing the phenotypes obtained by neuronal downregulation of *wg* or *fz2* using RNAi constructs (Fig. 4A-F). In all cases, the morphology of the NMJ resembled that observed in *Gαo*-overexpressing larvae (Fig. 3B,C). The morphological rescue was confirmed by quantitative analysis of bouton numbers (Fig. 4C,F). We further confirmed the epistasis between Gαo and Fz2 using genetic null alleles of *fz2*. Complete loss of Fz2 substantially alters the morphology of the NMJ and decreases bouton numbers (Fig. 4G,I). These phenotypes could be completely rescued by neuronal expression of *Gαo[Q205L]* (Fig. 4H,I). The same rescue of the *fz2* null could be achieved by *RNAi-sgg* (Fig. 4I).

Thus, Gαo acts as a (presumably immediate) transducer of Wg-Fz2 signaling in the NMJ. The similar efficiencies of the GTP- and GDP-loaded forms of Gαo in executing the Wg-Fz2 signal suggest that the molecular target(s) of Gαo in this signaling pathway does not discriminate between the two nucleotide states of the G protein.

### Ank2 physically binds to and acts downstream of Gαo in the *Drosophila* NMJ

**Fig. 5.**
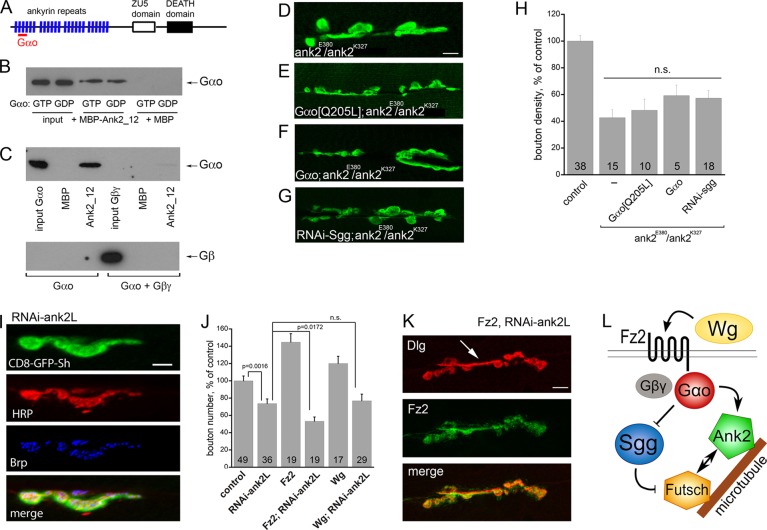
**Ank2 acts downstream from Gαo and physically interacts with it.** (A) Structure of Ank2, displaying the four ankyrin-repeat domains (each composed of six ankyrin repeats), the ZU5 (spectrin binding) and the DEATH domains. The Gαo binding site detected in the yeast two-hybrid screen is located between amino acids 47 and 123 (red bar). (B) Pull-down experiments between Gαo and truncated Ank2 (Ank2_12, consisting of the first 12 ankyrin repeats) confirm the yeast two-hybrid interaction. Gαo efficiently interacts with Ank2 regardless of the guanine nucleotide with which it is preloaded (GDP or GTPγS). Maltose-binding protein (MBP) is the negative control showing no interaction with Gαo. (C) The binding between Gαo and Ank2 is outcompeted by Gβγ: preincubation of Gαo with equimolar Gβγ drastically diminishes the amounts of Gαo competent to interact with Ank2_12; Gβγ is not pulled down by Ank2. The bottom western blot panel is intentionally overexposed to show that no Gβγ is pulled down by Ank2. (D-G) *Ank2* null reveals severe NMJ phenotypes (D) that are not rescued by overexpression of *Gαo[Q205L]* (E), *Gαo* (F) or *RNAi-sgg* (G). (H) Bouton density in *Ank2* null phenotypes. Data are shown as bouton number per length of NMJ, as percentage of control; n.s., not significant (*P*>0.05). (I) High magnification of *RNAi-Ank2* shows morphological defects similar to downregulation of Wg, Fz2 or Gαo. (J,K) Overexpression of Wg or Fz2 fails to rescue the reduced bouton formation (J; data shown as in Fig. 2A) and morphological abnormalities (K) of *RNAi-Ank2*. (K) Immunostaining for Dlg provides a postsynaptic marker, whereas Fz2-GFP marks the presynapse. Elongated tube-like, bouton-less staining is visible (arrow). (L) Model of microtubule cytoskeleton regulation during NMJ formation. The Wg-Fz2 ligand-receptor complex activates the heterotrimeric Go protein, releasing Gαo, which in turn inhibits the Sgg-containing destruction complex. As a result, Sgg-mediated phosphorylation of Futsch is decreased. Futsch, in parallel, interacts with Ank2, the latter additionally being under direct control by Gαo. This combined action on microtubule-binding proteins coordinately regulates the microtubule cytoskeleton, as required for synaptic remodeling. Scale bars: 10 µm in D,K; 5 µm in I.

To identify potential Gαo target proteins, we performed a yeast two-hybrid screen with a *Drosophila* head cDNA library as prey and Gαo as bait (Kopein and Katanaev, 2009). We identified three clones of Ank2 interacting with Gαo with high confidence. The interaction site could be narrowed to amino acids 47-123 of Ank2 (Fig. 5A; see Materials and Methods). In order to confirm the Gαo-Ank2 interaction and to investigate its dependence on guanine nucleotides, we bacterially expressed and purified a truncated maltose-binding protein (MBP)-tagged Ank2 construct (Ank2_12) that consisted of the first 12 ankyrin repeats containing the Gαo binding site (see supplementary material Fig. S3A for characterization of the resulting recombinant protein). We additionally purified highly active recombinant Gαo (Kopein and Katanaev, 2009). In the pull-down experiments, we found that Gαo and Ank2_12 efficiently interacted with each other, supporting the yeast two-hybrid data (Fig. 5B). The GDP- and GTPγS-loaded forms of Gαo were equally efficient in Ank2 binding, expanding the list of Gαo target proteins that do not discriminate between the two nucleotide forms of this G protein (Katanaev, 2010). Importantly, preincubation of Gαo with Gβγ dramatically reduced the amounts of Gαo pulled down by Ank2_12 (Fig. 5C, top). Furthermore, the small amounts of Gαo still interacting with Ank2_12 in this experiment remained Gβγ free, as no Gβγ was detected in Ank2 pull-downs (Fig. 5C, bottom). Thus, Ank2 behaves as a true effector of Gαo, interacting with the monomeric Gβγ-free form of this G protein.

The described (Koch et al., 2008; Pielage et al., 2008; see also Fig. 5D) phenotypes of *Ank2* mutants resemble those that we see upon RNAi-mediated presynaptic downregulation of *Gαo*, *fz2* and *wg*. To test whether Ank2 is epistatic to Wg-Fz2-Gαo signaling, we overactivated this pathway at different levels in the *Ank2* null background. Overexpression of *Gαo* or *Gαo[Q205L]* or downregulation of *sgg* failed to rescue the bouton morphology of the *Ank2* nulls (Fig. 5D-G), and the bouton density remained severely decreased (Fig. 5H), suggesting that Ank2 is epistatic to both Gαo and Sgg in synapse formation. However, Gαo could still localize to the NMJ despite Ank2 absence (supplementary material Fig. S3C), demonstrating that Ank2 does not merely control Go localization in the NMJ.

We also expressed RNAi against *Ank2L* (Pielage et al., 2008) with *OK371-Gal4*, producing morphological defects similar to those resulting from downregulation of *wg*/*fz2*/*Gαo* (Fig. 5I). Overexpression of *wg* or *fz2* in the *RNAi-Ank2L* background failed to restore or improve the synaptic morphology and bouton numbers of *Ank2* downregulation (Fig. 5J,K). Fz2 faithfully localizes to the NMJ despite reduced Ank2 levels (Fig. 5K; supplementary material Fig. S2G), again arguing that Ank2 does not simply regulate the localization of Wg-Fz2-Gαo signaling components. Altogether, Ank2 appears to act downstream of the Wg-Fz2-Gαo pathway.

As Ank2 has been shown to regulate bouton stability (Hortsch et al., 2002; Koch et al., 2008; Pielage et al., 2008), we next analyzed the extent of synaptic retractions in *Ank2* mutants with or without activation of Gαo. Loss of the microtubule-binding protein Futsch is considered as the first step of synaptic retraction, followed by loss of cytoplasmic proteins such as Synapsin (Pielage et al., 2008). In accordance with previous studies (Koch et al., 2008; Pielage et al., 2008), we observed that ∼40% of the *Ank2^−/−^* boutons lost Synapsin staining and ∼60% lost Futsch (supplementary material Fig. S3D,F,H). As expected, expression of *Gαo[Q205L]* in the *Ank2^−/−^* NMJs failed to restore synaptic stability when evaluated at the level of Synapsin or Futsch (supplementary material Fig. S3E,G,H). Thus, Gαo cannot rescue synapse stability in the absence of Ank2, confirming that Ank2 is epistatic to the Wg-Fz2-Gαo pathway.

We next analyzed presynaptic abnormalities in NMJs with reduced Gαo and found that ∼8% of *Gαo* mutant boutons and 5.4% of the *RNAi-Gαo* boutons are completely devoid of Ank2 immunostaining [supplementary material Fig. S3I; 7.91±2.71% (*n*=18) and 5.41±1.73% (*n*=23), respectively, as compared with 0.73±0.30% (*n*=31) in wild-type NMJs (mean±s.e.m.); *P*=0.0012 and *P*=0.0033, respectively]. Reciprocally, in the absence of Ank2, overactivation of Gαo induces a significant number of ghost boutons and neuronal processes [bouton-like structures and interconnecting processes containing presynaptic HRP staining but lacking postsynaptic CD8-GFP-Sh (Ataman et al., 2006)] (supplementary material Fig. S3J,K); such structures are rarely visible in other genotypes (Ataman et al., 2006). Thus, it can be suggested that the Wg-Fz2-Gαo pathway recruits Ank2 to build a synapse, and in the absence of the latter the synapse does not form properly.

### Gαo-ankyrin interaction is conserved in the mammalian neurite outgrowth pathway

As an independent means of proving the mechanistic relationship between Gαo and ankyrins, and to show that this interaction is of importance beyond the *Drosophila* NMJ, we turned to the well-characterized neurite outgrowth pathway in mouse neuroblastoma N2a cells.

**Fig. 6.**
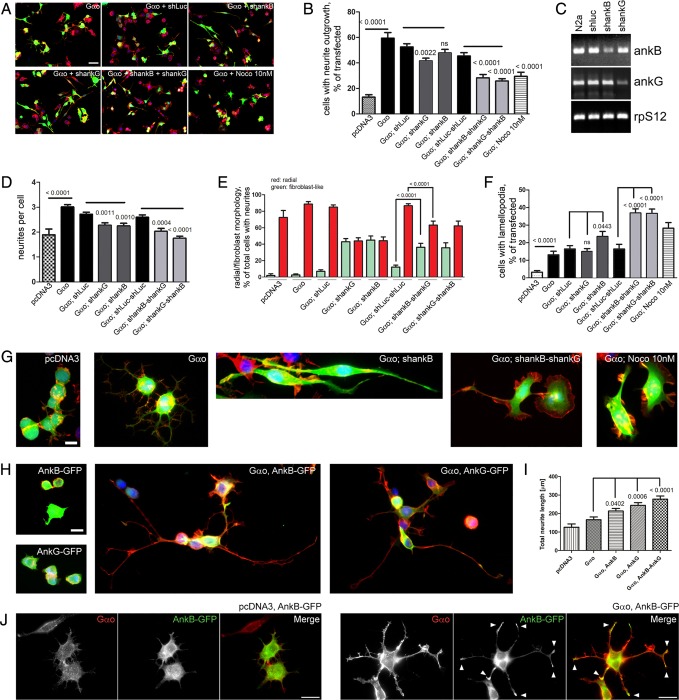
**Gαo-mediated neurite outgrowth and neuronal morphology in N2a cells require AnkB and AnkG.** (A) Overexpression of Gαo stimulates the formation of neurites in parental mouse N2a cells and in cells stably transfected with control shRNA (shluc). Permanent shRNA-induced downregulation of *AnkB* (shankB) or *AnkG* (shankG) results in the formation of elongated fibroblast-like cells, increases lamellopodia formation and slightly reduces the percentage of cells growing neurites and the number of neurites per cell. Transient ankyrin double knockdowns achieved by transfection of the shankB and shankG stable cell lines with the shankG and shankB plasmids, respectively, strongly increase the effects observed in single knockdowns. Treatment of Gαo-overexpressing N2a cells with Nocodazole (Noco) mimics the ankyrin double-knockdown phenotypes. Co-expression of EGFP (green) marks transfected cells and staining with phalloidin-Rhodamine (red) and DAPI (blue) is used to visualize F-actin and nuclei, respectively. (B) Quantification of the effects of Gαo overexpression on neurite outgrowth as compared with control transfected (pcDNA3) N2a cells, in shRNA stably transfected cell lines and in the presence of 10 nM Nocodazole. Data represent mean±s.e.m.; horizontal black lines indicate groups of statistical analysis and *P*-values are given above each bar (ns, not significant). (C) RT-PCR analysis shows the reduction in *AnkB* and *AnkG* expression in shRNA stably transfected N2a cells. Expression of the ribosomal protein S12 gene (*Rps12*) served as control. (D-F) Quantification of effects on the number of neurites per cell (D), cell morphology (E) and lamellopodia formation (F) of overexpression of Gαo in parental and shRNA-treated N2a cells. Data representation and statistical analysis are as in B. (G) Representative images of control transfected (pcDNA3) N2a cells and Gαo overexpression in parental as well as in single and double AnkB and AnkG knockdowns. Nocodazole treatment mimics the effects of Gαo overexpression in ankyrin double knockdowns. (H) Representative images of N2a cells overexpressing EGFP-tagged AnkB or AnkG show a substantial increase in the length of neurites upon co-expression with *Gαo*, but not alone. Fluorescence as in A. (I) Quantification of total neurite length in H. Data representation and statistical analysis are as in B. (J) Overexpression of *Gαo* induced the local accumulation of AnkB-GFP at neurite tips (arrowheads), which is not observed in control cells transfected with AnkB-GFP alone. Red fluorescence indicates Gαo immunostaining. Scale bars: 20 µm in A; 10 µm in G,H,J.

As previously reported (Jordan et al., 2005), we find that overexpression of *Gαo* induces strong neurite outgrowth in N2a cells (Fig. 6A,B), with ∼60% of cells forming neurites. N2a cells express both neuronal mammalian ankyrins: *AnkB* and *AnkG* (*Ank2* and *Ank3* – Mouse Genome Informatics) (Fig. 6C) (Santuccione et al., 2013). We downregulated *AnkB*, *AnkG* or both using shRNA constructs (Fig. 6C), and investigated whether Gαo was still capable of inducing neurite outgrowth in these mutant backgrounds. The overall number of N2a cells with neurite outgrowth, as well as the number of neurites per cell induced by Gαo, were decreased in *AnkB* and *AnkG* single knockdowns, and further decreased in double knockdowns (Fig. 6A,B,D).

However, the most dramatic effect of *AnkB*/*G* knockdown on Gαo-induced neurite outgrowth was seen at the level of overall cell morphology (Fig. 6E-G). Whereas *Gαo*-overexpressing cells (as well as N2a cells spontaneously producing neurites) possessed a radial morphology, with several neurites undergoing outgrowth in multiple directions (Fig. 6E,G), *Gαo* overexpression in *AnkB* and *AnkG* single knockdowns induced a very characteristic bilateral, fibroblast-like morphology (Fig. 6E,G), which often additionally included the formation of lamellopodia (Fig. 6G). Remarkably, the double knockdowns further increased the number of cells that were massively producing lamellopodia instead of neurites (Fig. 6F,G). It appears that the lamellopodial phenotype of *Gαo*-overexpressing, *AnkB*/*G* double-knockdown cells is a more severe manifestation of the fibroblast-like morphology seen in *Gαo*-overexpressing, *AnkB* or *AnkG* single-knockdown cells (Fig. 6E,F). By contrast, *AnkB*/*G* knockdowns in control cells do not change in cellular appearance (supplementary material Fig. S4A). As an independent means to induce neurite outgrowth, we overexpressed MARK2 (also known as PAR1b) (Biernat et al., 2002) and found that the resulting phenotype was unaffected by the double knockdown of *AnkB* and *AnkG* (supplementary material Fig. S4B,C), indicating that akyrins are specifically required for the Gαo-mediated neurite outgrowth pathway.

Thus, reduction in ankyrin levels dramatically alters the ability of Gαo to induce neurite outgrowth in neuronal cells and further changes the cytoskeletal response to Gαo – from neurite production to lamellopodial protrusion. We hypothesized that, in the absence of AnkB/G, the Gαo-responsive cellular program switches from the regulation of microtubules to the actin cytoskeleton. To test this, we treated the *Gαo*-overexpressing cells with different concentrations of nocodazole, which is a microtubule-depolymerizing agent known to impair neurite outgrowth (Heidemann et al., 1985). Remarkably, low nocodazole concentrations could mimic the effect of *AnkB*/*G* double knockdown in *Gαo*-overexpressing cells: the ability of Gαo to induce neurite outgrowth was reduced, with a concomitant increase in the number of lamellopodial cells (Fig. 6F,G; supplementary material Fig. S4D,E).

Next, we examined the effects of co-overexpression of *Gαo* with EGFP-tagged *AnkB* and/or *AnkG*. Notably, co-overexpression of *Gαo* and *AnkB*, *AnkG* or both induced a substantial increase in the total neurite length compared with *Gαo* overexpression alone (Fig. 6H,I), whereas the number of cells displaying neurites and the number of neurites per cell were unaffected (supplementary material Fig. S4F,G). As overexpression of *AnkB* and/or *AnkG* did not induce neurite outgrowth (Fig. 6H), these data further support the functional relationship between Gαo and ankyrins. Interestingly, AnkB (but not AnkG) significantly accumulates at the tips of neurites in *Gαo*-overexpressing cells, but not at spontaneously formed neurites in control N2a cells or at neurites induced by MARK2 co-expression (Fig. 6J; supplementary material Fig. S4H-J). These results indicate that Gαo activity is required to recruit AnkB to the growing neurite tips.

We conclude that the Gαo-Ank interaction is conserved from *Drosophila* to mammalian cells, and that this interaction is crucial for the ability of Gαo to regulate the neuronal microtubule cytoskeleton.

## DISCUSSION

Synaptic plasticity underlies learning and memory. Both in invertebrates and vertebrates, activation of Wnt signaling is involved in several aspects of synapse formation and remodeling (Budnik and Salinas, 2011), and defects in this pathway may be causative of synaptic loss and neurodegeneration (Inestrosa and Arenas, 2010). Thus, understanding the molecular mechanisms of synaptic Wnt signaling is of fundamental as well as medical importance. The *Drosophila* NMJ is a powerful model system with which to study glutamatergic synapses (Collins and DiAntonio, 2007), and the Wnt pathway has been widely identified as one of the key regulators of NMJ formation (Packard et al., 2002; Mathew et al., 2005; Miech et al., 2008; Korkut et al., 2009; Mosca and Schwarz, 2010).

Here, we provide important mechanistic insights into Wnt signal transduction in the NMJ, identifying the heterotrimeric Go protein as a crucial downstream transducer of the Wg-Fz2 pathway in the presynapse. We further demonstrate that Ank2, a known player in the NMJ (Koch et al., 2008; Pielage et al., 2008), is a target of Gαo in this signaling.

We find that the α subunit of Go is strongly expressed in the presynaptic cell, and that under- or overactivation of this G protein leads to neurotransmission and behavioral defects. At the level of NMJ morphology, we find that presynaptic downregulation or Ptx-mediated inactivation of *Gαo* recapitulates the phenotypes obtained by similar silencing of *wg* and *fz2*. These data confirm that presynaptic Wg signaling, in addition to the Wg pathway active in the muscle (Mathew et al., 2005; Mosca and Schwarz, 2010), is crucial for proper NMJ formation (Miech et al., 2008), and that Go is required for this process. Furthermore, neuronal Gαo overexpression can rescue the *wg* and *fz2* loss-of-function phenotypes, demonstrating that, as in other contexts of Wnt/Fz signaling (Katanaev et al., 2005; Katanaev and Tomlinson, 2006a; Purvanov et al., 2010), Go acts as a transducer of Wg/Fz2 in NMJ formation. In contrast to its evident function and clear localization in the presynapse, Gαo localization on the muscle side of the synapse is much less pronounced or absent. Unlike Gαo, the main *Drosophila* Gβ subunit is strongly expressed in both the pre- and postsynapse. Thus, a heterotrimeric G protein other than Go might be involved in the postsynaptic Fz2 transduction, as has been implicated in Fz signaling in some other contexts (Egger-Adam and Katanaev, 2008; Koval and Katanaev, 2011; von Maltzahn et al., 2012; Nichols et al., 2013).

A recent study proposed a role for Gαo downstream of the octopamine receptor Octβ1R (Koon and Budnik, 2012). This signaling was proposed to regulate the acute behavioral response to starvation both on type II NMJs (octapaminergic) and on the type I NMJs (glutamatergic) studied here. In contrast to our observations, downregulation of Gαo in these NMJs was proposed to increase, rather than decrease, type I bouton numbers (Koon and Budnik, 2012). We suspect that the main reason for the discrepancy lies in the Gal4 lines used. The *BG439-Gal4* and *C380-Gal4* lines of Koon and Budnik are poorly characterized and, unlike the well-analyzed pan-neuronal *elav-Gal4* (Luo et al., 1994) and motoneuron-specific *OK371-Gal4* (Mahr and Aberle, 2006) and *D42-Gal4* (Parkes et al., 1998) driver lines used in our study, might mediate a more acute expression. In this case, our study reflects the positive role of Gαo in the developmental formation of glutamatergic boutons, as opposed to a role in acute fine-tuning in response to environmental factors as studied by Koon and Budnik (2012).

Postsynaptic expression of *fz2* was found to fully rescue *fz2* null NMJs (Mathew et al., 2005) (supplementary material Fig. S1Q). Here, we find that presynaptic knockdown of Fz2 (and other components of Wg-Fz2-Gαo signaling) recapitulates *fz2* null phenotypes, whereas presynaptic overactivation of this pathway increases bouton numbers; furthermore, presynaptic overexpression of *fz2* or *Gαo* rescues the *fz2* nulls, just as postsynaptic overexpression of *fz2* does. Our data thus support a crucial role for presynaptic Wg-Fz2-Gαo signaling in NMJ formation. Interestingly, both pre- and postsynaptic re-introduction of Arrow, an Fz2 co-receptor that is normally present both pre- and postsynaptically, as is Fz2 itself, can rescue *arrow* mutant NMJs (Miech et al., 2008). Thus, it appears that the pre- and postsynaptic branches of Fz2 signaling are both involved in NMJ development. A certain degree of redundancy between these branches must exist. Indeed, wild-type levels of Fz2 in the muscle are not sufficient to rescue the bouton defects induced by presynaptic expression of *RNAi-fz2* (Fig. 2A,B,G), yet overexpression of *fz2* in the muscle can restore the bouton integrity of *fz2* nulls (supplementary material Fig. S1Q) (Mathew et al., 2005). One might hypothesize that postsynaptic Fz2 overexpression activates a compensatory pathway – such as that mediated by reduction in laminin A signaling (Tsai et al., 2012) – that leads to restoration in bouton numbers in *fz2* mutants. Our data showing that the targeted downregulation of Fz2 in the presynapse is sufficient to recapitulate the *fz2* null phenotype underpin the crucial function of presynaptic Fz2 signaling in NMJ formation.

We find that downregulation of *Ank2* produces NMJ defects similar to those of *wg*, *fz2* or *Gαo* silencing. However, *Ank2* mutant phenotypes appear more pronounced, indicating that Wg-Fz2-Gαo signaling might control a subset of Ank2-mediated activities in the NMJ. Ank2 was proposed to play a structural role in NMJ formation, binding to microtubules through its C-terminal region (Pielage et al., 2008). However, since the C-terminal region was insufficient to rescue *Ank2L* mutant phenotypes (Pielage et al., 2008), additional domains are likely to mediate Ank2 function through binding to other proteins. We demonstrate here in the yeast two-hybrid system and in pull-down experiments that the ankyrin repeat region of Ank2 physically binds Gαo, suggesting that the function of Ank2 in NMJ formation might be regulated by Wg-Fz2-Gαo signaling. Indeed, epistasis experiments place Ank2 downstream of Gαo in NMJ formation.

Upon dissociation of the heterotrimeric Go protein by activated GPCRs such as Fz2, the liberated Gαo subunit can signal to its downstream targets both in the GTP- and GDP-bound state (the latter after hydrolysis of GTP and before re-association with Gβγ) (Katanaev, 2010). The free signaling Gαo-GDP form is predicted to be relatively long lived (Katanaev and Chornomorets, 2007), and a number of Gαo target proteins have been identified that interact equally well with both of the nucleotide forms of this G protein (Kopein and Katanaev, 2009; Egger-Adam and Katanaev, 2010; Purvanov et al., 2010; Lin and Katanaev, 2013; Lin et al., 2014). In the context of NMJ formation, we find that Gαo-GTP and -GDP are efficient in the activation of downstream signaling, and identify Ank2 as a binding partner of Gαo that interacts with both nucleotide forms. The importance of signaling by Gα-GDP released from a heterotrimeric complex by the action of GPCRs has also been demonstrated in recent studies of mammalian chemotaxis, planar cell polarity and cancer (Ezan et al., 2013; Kamakura et al., 2013; Lin et al., 2014).

Gαo[G203T], which largely resides in the GDP-binding state owing to its reduced affinity for GTP, might be expected to act as a dominant-negative. However, in canonical Wnt signaling, regulation of asymmetric cell division as well as in planar cell polarity (PCP) signaling in the wing, Gαo[G203T] displays no dominant-negative activity but is simply silent (Katanaev et al., 2005; Katanaev and Tomlinson, 2006a), whereas in eye PCP signaling this form acts positively but is weaker than other Gαo forms (V.L.K. and A. Tomlinson, unpublished observations). Biochemical characterization of the mammalian Gαi2[G203T] mutant revealed that it can still bind Gβγ and GTP, but upon nucleotide exchange Gαi2[G203T] fails to adopt the activated confirmation and can further lose GTP (Inoue et al., 1995). Our biochemical characterization confirms that Gαo[G203T] still binds GTP (supplementary material Fig. S2C). Interestingly, Gαi2[G203T] inhibited only a fraction of Gαi2-mediated signaling (Winitz et al., 1994), suggesting that the dominant-negative effects of the mutant are effector specific. Thus, we infer that a portion of Gαo[G203T] can form a competent Fz2-transducing complex, and a portion of overexpressed Gαo[G203T] resides in a free GDP-loaded form that is also competent to activate downstream targets – Ank2 in the context of NMJ formation.

Our experiments place Ank2 downstream of Gαo and also of Sgg (GSK3β). It remains to be investigated whether Ank2 can directly interact with and/or be phosphorylated by Sgg. Meanwhile, we propose that the microtubule-binding protein Futsch might be a linker between Sgg and Ank2. Futsch is involved in NMJ formation and is placed downstream of Wg-Sgg signaling, being the target of phosphorylation and negative regulation by Sgg as the alternative target to β-catenin, which is dispensable in Wg NMJ signaling (Hummel et al., 2000; Roos et al., 2000; Franco et al., 2004; Gogel et al., 2006; Miech et al., 2008). Abnormal Futsch localization has been observed in *Ank2* mutants (Pielage et al., 2008). In *Drosophila* wing and mammalian cells in culture, Gαo acts upstream of Sgg/GSK3β (Katanaev et al., 2005; Liu et al., 2005). Cumulatively, these data might suggest that the Wg-Fz2-Gαo cascade sends a signal to Futsch through Sgg, parallel to that mediated by Ank2 (Fig. 5L).

The importance of the Gαo-Ank2 interaction for *Drosophila* NMJ development is corroborated by our findings in mammalian neuronal cells, where we demonstrate that the ability of Gαo to induce neurite outgrowth is critically dependent on AnkB and AnkG. Knockdown of either or both ankyrin reduces neurite production. Remarkably, upon AnkB/G downregulation, Gαo switches its activity from the induction of microtubule-dependent processes (neurites) to actin-dependent protrusions (lamellopodia). Furthermore, Gαo recruits AnkB to the growing neurite tips. These data demonstrate that the Gαo-ankyrin mechanistic interactions are conserved from insects to mammals and are important for control over the neuronal tubulin cytoskeleton in the context of neurite growth and synapse formation. The novel signaling mechanism that we have uncovered (Fig. 5L) might thus be of general applicability in animal nervous system development and function.

## MATERIALS AND METHODS

### Fly stocks

Fly lines are described in supplementary material Methods. Fly crosses were performed at 25°C.

### Immunostaining and microscopy analysis of NMJs

Wandering third instar larvae were dissected in PBS as described (Brent et al., 2009) before fixation and immunostaining using the antibodies described in supplementary material Methods. NMJs of muscle 6/7 in segment 2-4 were analyzed in all experiments. Maximally, two segments per animal were analyzed. NMJs were imaged with a Zeiss LSM 510 or LSM710 confocal microscope. For further details see supplementary material Methods.

### Electrophysiology and muscle contraction

ChR2-mediated stimulation of synaptic potentials was performed as described (Schroll et al., 2006; Hornstein et al., 2009) and intracellular potentials were recorded in body wall muscles 6/7 (for details see supplementary material Methods).

### Yeast two-hybrid screen, pull-down assay and GTP-binding assay

The yeast two-hybrid screen, biological significance score and analysis of the Gαo-interacting region in Ank2 were performed as described (Formstecher et al., 2005; Kopein and Katanaev, 2009). The first 12 ankyrin repeats of Ank2 (Ank2_12) were cloned into pMAL-c2x (New England BioLabs). The MBP-tagged Ank2_12 and MBP alone were bacterially expressed and purified. Recombinant *Drosophila* His_6_-Gαo and His_6_-Gαo[G203T] were purified in parallel and pull-downs and GTP-binding assays were performed as previously described (Kopein and Katanaev, 2009; Koval et al., 2010). Further details are provided in supplementary material Methods.

### Mouse cell culture and neurite outgrowth assay

Mouse neuroblastoma N2a cells were cultured in MEM supplemented with 10% FCS, L-glutamine and penicillin/streptomycin (all from Gibco, Life Technologies). Vector transfections were carried out with X-tremeGENE 9 (Roche) according to the manufacturer's instructions. Permanent AnkB or AnkG depletion in N2a cells was achieved using the pRetroSuper vector (Oligoengine). For the analysis of neurite outgrowth, cells were transfected for 24 h, trypsinized and seeded on poly-L-lysine-coated coverslips for an additional 24 h to allow neurite formation. For Nocodazole (Sigma-Aldrich) treatment, transfected N2a cells were allowed to adhere on coverslips for 6 h before incubation for an additional 18 h with Nocodazole. Cells were finally fixed with 4% paraformaldehyde, stained with phalloidin-Rhodamine (Molecular Probes, Life Technologies) and DAPI (Sigma-Aldrich) or anti-Gαo antibody and mounted for microscopy analysis. For further details see supplementary material Methods.

### Statistical analysis

Statistical analysis was performed with SAS JMP 7 and GraphPad Prism 5. Data are presented as mean±s.e.m. *P*-values were obtained by Student's *t*-test.

## Supplementary Material

Supplementary Material
