# Supplementary material for: Heterotrimeric Go protein links Wnt-Frizzled signaling with ankyrins to regulate the neuronal microtubule cytoskeleton
Source: Development. 2014 Sep;141(17):3399–409. doi: 10.1242/dev.106773 (PMC4199127; doi:10.1242/dev.106773)
Supplement: Supplementary Material [file supp_141_17_3399__index.html]

Heterotrimeric Go protein links Wnt-Frizzled signaling with ankyrins to regulate the neuronal microtubule cytoskeleton — Supplementary Material 

# Heterotrimeric Go protein links Wnt-Frizzled signaling with ankyrins to regulate the neuronal microtubule cytoskeleton

## DEV106773 Supplementary Material

**Files in this Data Supplement:**

- **Supplementary Material**
