# Supplementary material for: Heterotrimeric Go protein links Wnt-Frizzled signaling with ankyrins to regulate the neuronal microtubule cytoskeleton
Source: Development. 2014 Sep;141(17):3399–409. doi: 10.1242/dev.106773 (PMC4199127; doi:10.1242/dev.106773)
Supplement: Supplementary Material [file supp_141.17.3399_DEV106773.pdf]

SUPPLEMENTARY METHODS

Fly stocks

The following lines used were: *OK371-Gal4* (Mahr and Aberle, 2006); *CD8-GFP-Sh* (Zito et al., 1999); *UAS-RNAi-ank2L* (Pielage et al., 2008); *UAS-GFP-Wg* (Pfeiffer et al., 2002); *UAS-Ptx*, *UAS-Gαo*, *UAS-Gαo[Q205L]*, *UAS-Gαo[G203T]* (Katanaev et al., 2005); *UAS-Fz2* (Chen et al., 2004); *UAS-Fz* (Strapps and Tomlinson, 2001); *UAS-wg-GFP* (Pfeiffer et al., 2002); *omb-Gal4* (Lecuit et al., 1996). The following lines were from the Vienna *Drosophila* RNAi Center (Dietzl et al., 2007): *UAS-RNAi-fz2* (VDRC#44391), *UAS-RNAi-Gαo* (#19124 and #110552 were used with identical results), *UAS-RNAi-wg* (#13351), *UAS-RNAi-ank2* (#26121), *UAS-RNAi-sgg* (#7005). *Df(3L)ED4782* deficiency (Hummel et al., 2000), *UAS-ChR2* (Schroll et al., 2006), *elav-Gal4*, D42-Gal4, *BG487-Gal4*, *GMR-Gal4* and *UAS-myr-mRFP* were from the Bloomington Stock Center. The *fz2* mutant condition was $fz2^{C1}$/ *Df(3L)ED4782* following Mathew et al. (2005). The $ank2^{E380}$ and $ank2^{K327}$ alleles (Koch et al., 2008) were used in the transheterozygote combination to analyze the *Ank2* mutant phenotypes. Although *Gαo* mutant alleles are embryonic lethal (Fremion et al., 1999; Katanaev et al., 2005), we could obtain third instar larvae of the transheterozygous genotype $G\alpha o^{007}/G\alpha o^{EXGO\text{-}UK}$. The first allele is a hypomorph (Fremion et al., 1999), whereas the second is a small deletion in the region (gift from A. Tomlinson). The transheterozygous larvae emerged from the genetic cross at a frequency of 23% (expected frequency 33%) but developed 1-2 days later than their heterozygous siblings; they died during early pupal stages. For the *Gαo* rescue experiments, $G\alpha o^{EXGO\text{-}UK}$ was recombined with *OK371-Gal4*; the presence of the driver in the recombinant was confirmed by crossing to *UAS-myr-mRFP*; the presence of the mutation was confirmed by lethality over the parental and other *Gαo* alleles. The muscle size of the heterozygous larvae was somewhat reduced compared with control larvae (Fig. S2F). All crosses were performed at 25°C.

Antibodies and immunohistochemistry

Wandering third instar larvae were dissected in PBS as described (Brent et al., 2009), fixed in 3.7% formaldehyde/PBS or, in the case of anti-Wg staining, in Bouins fixative (Reactives RAL) for 15 min and washed three times in PBS for 10 min. The dissected larvae were incubated in PBS containing 0.05% Triton X-100 (PBT) + 5% normal goat serum (NGS) for at least 30 min at room temperature. Primary antibodies were diluted in PBT plus 5% NGS and incubated at room temperature for 2 h or at 4°C overnight. The following primary antibodies were used: Cy3-coupled goat anti-HRP (123-165-021, Jackson ImmunoResearch) at 1:200; rabbit anti-Wg (Reichsman et al., 1996) at 1:300; rabbit anti-Fz2 (Packard et al., 2002) at 1:10,000; rabbit anti-Gβ13F (Schaefer et al., 2001) at 1:250; and anti-Ank2XL (Koch et al., 2008) at 1:500; mouse anti-Brp (nc82), anti-Dlg (4F3), anti-Futsch (22C10) and anti-Synapsin (3C11) (all at 1:100; Developmental Studies Hybridoma Bank); rabbit anti-Gαo at 1:100 (Merck, #371726, raised against the C-terminal decapeptide of human Gαo and Gαi3). The specificity of these anti-Gαo antibodies to recognize *Drosophila* Gαo but not Gαi was confirmed in wing imaginal discs of *omb-Gal4; UAS-Gαo* and *omb-Gal4; UAS-Gαi* larvae (Fig. S1E,F). The efficiency of these overexpression lines had been tested previously (Katanaev and Tomlinson, 2006); wing discs were immunostained as described (Katanaev et al., 2005). Additionally, the specificity of these antibodies (1:1000) to *Drosophila* Gαo but not Gαi was proven by western blots of head extracts (Kopein and Katanaev, 2009) from wild-type, *UAS-Gαo; GMR-Gal4* and *UAS-Gαi; GMR-Gal4* flies (Fig. S1G). Gαi, migrating lower than Gαo on SDS-PAGE, was undetected in *Drosophila* heads without overexpression, but was efficiently overexpressed with the *UAS-Gαi* construct as detected by polyclonal anti-Gαi antibodies (Merck, #371723, raised against the C-terminal decapeptide of human Gαi1 and Gαi2; used at 1:1000); these antibodies also recognized *Drosophila* Gαo (Fig. S1G). Additional rabbit polyclonal antibodies against *Drosophila* Gαo

were raised using the recombinant protein purified from bacteria (Kopein and Katanaev, 2009); the antiserum was used at 1:100 for immunostaining. Specificity of this antiserum was confirmed by western blots on *Drosophila* head extracts, as well as by immunostaining of wing imaginal discs. Secondary antibodies were HRP labeled for western blots (1:4000) or Cy3- and Cy5-labeled in immunostaining (1:400 in PBT, 2h incubation at room temperature). The preparations were mounted in Vectashield (Vector Labs), dorsal side up.

Microscopy and analysis of NMJs

The well-characterized NMJs of muscle 6/7 in segment 2-4 were analyzed in all experiments. Maximally, two segments per animal (e.g. segment A3 and A4, both in the same hemisphere, or both segments A3 in the two hemispheres) were analyzed. NMJs were imaged with a confocal microscope (Zeiss LSM 510 or Zeiss LSM710). For statistical analysis, one optical slice with a thickness of 2.3 µm was taken with a 20x or a 25x objective in the optical plane of each NMJ and the boutons were measured manually with the help of the program AxioVision 4.7 (Zeiss). A bouton was identified by the CD8-GFP-Sh, anti-Dlg and/or anti-HRP staining as a circular or slightly oval structure with clear borders, connected by neurites to the neighboring bouton; all these methods resulted in identical bouton quantifications. Type 1b boutons were distinguished from type Is by more intense anti-Dlg staining and their larger size (Packard et al., 2002). Bouton number values are depicted as percentage of the respective control. The length of the NMJ was measured from the first to the last bouton along the synaptic cleft and all side branches on the muscle surface with more than three boutons were measured and added to the total length of the NMJ. The lengths of the NMJ slightly varied depending on the phenotype, in agreement with (Mathew et al., 2005) (Fig. S2E).

To confirm that the *OK371-Gal4/UAS-RNAi* system was efficient to downregulate Gαo, Wg and Fz2, respective immunostainings of wild-type and *RNAi*-expressing NMJs were

performed in parallel. NMJs were imaged with a LSM710 (Zeiss) confocal microscope using identical settings for all images. Quantification of the fluorescence was performed with ImageJ (NIH). The presynaptic cell was outlined with the freehand selection tool following the borders of the staining and the mean value of the fluorescence of this area was measured with the measure tool. A noticeable downregulation in the levels of the respective proteins was achieved (Fig. S1I,M,O), and quantification revealed a ~50% decrease in anti-Gαo/Wg/Fz2 staining in the NMJ (Fig. S1J,N,P). However, this is likely to be a gross underestimation of the efficiency of the RNAi-mediated downregulation: using a pan-neuronal driver (*elav-Gal4*), we find a comparable decrease in anti-Gαo immunostaining (Fig. S1K), but in western blots on whole-head extracts of the control versus the *RNAi-Gαo* constructs, a dramatic decrease in Gαo levels could be seen (Fig. S1L). Mouse anti-tubulin (Sigma, 1:2500) staining served as loading control.

Electrophysiology and muscle contraction

ChR2-mediated stimulation of synaptic potentials was performed as described (Schroll et al., 2006; Hornstein et al., 2009). Larvae expressing ChR2 in motoneurons using the driver *OK371-Gal4* were grown on standard corn food supplemented with 1 mM *all-trans*-retinal (Sigma) at 25°C in the dark. Wandering third instar larvae were dissected in cold Ca^{2+}-free HL-3 saline (Zhang and Stewart, 2010) and washed three times in cold HL-3 supplemented with 1.5 mM $CaCl_2$ before performing the measurements in same buffer. Intracellular potentials were recorded in body wall muscles 6/7 using a pipette with a resistance of 15-30 MΩ when filled with 1 M KCl. To evoke single action potentials, animals were stimulated by a 20 ms light pulse of 470 nm using a high-power LED placed 10 cm from the larvae (light pulse triggered at 1.2V, Thorlabs) controlled by Chart Master software (HEKA). Electrophysiological signals were pre-amplified (without filtering) using the LPF-8 signal conditioner (Warner Instruments) and the 50 Hz noise was reduced using the HumBug (Quest scientific) noise reducer. Finally, analog signals were

measured using a KS-700 amplifier (World Precision Instruments) then digitized using LIH8+8 (HEKA). Data were acquired using the Chart Master software at a sampling frequency of 20 kHz. The data were low-pass filtered at 2 kHz before analysis of EJPs and mEJPs with the Mini analysis program (Synaptosoft). The threshold for detection of peaks was set to 0.3 mV. For analysis only muscles with a resting potential more negative than –46 mV were used. Note that the optogenetic measurements used here and the traditional electrophysiological recordings produce identical EJP amplitudes when measured side-by-side (Pulver et al., 2011) (note also that, in this particular work, utilizing the same *OK371-Gal4* driver as used by us, the EJP amplitude in the wild-type is measured as ~12.5 mV, very similar to our measurement of 11 mV; see Fig. 1H). Furthermore, when performing our own optogenetic measurements, we sometimes (rarely) observed non-stimulated, spontaneous action potentials which were of the same amplitude as the light-induced ones (such an example is shown on Fig. S1T).

For the locomotion test, third instar larvae were placed on a 1% agarose plate and allowed to adjust for 1 min. The number of whole body contractions per minute was counted.

Yeast two-hybrid screen

Isoform II of *Drosophila* Gαo was used as the bait, and the cDNA library from *Drosophila* head was used as the prey in the screening custom-performed by Hybrigenics (Paris, France). Fifty four million clones were screened and analyzed as described (Kopein and Katanaev, 2009). The three clones of Ank2 representing partial open reading frames each had the high confidence interaction score]biological significance score (Formstecher et al., 2005)] (B, *E*-value < 1e–5). The Gαo-interacting region in Ank2 was determined as described (Formstecher et al., 2005; Kopein and Katanaev, 2009).

Biochemistry

We amplified the first 12 ankyrin repeats of Ank2 from cDNA clone RE55168 (*Drosophila* Genomics Resource Center, EST collection) using primers 5'-GGGCATGCATGGCCCAGTTTGTGACC-3' and 5'-CCGGTACCGGCACTAATGCTGGCACC-3' and inserted the fragment into pMAL-c2x (New England Biolabs). The MBP-tagged protein was expressed in TOP10F' cells (Invitrogen). Transformed cells were grown at 37°C until OD_{600} = 0.7, cooled to 17°C before induction with 0.1 mM IPTG and subsequent growth overnight at 17°C, and then harvested by 15 min centrifugation at 4000 ***g***. The pellet was resuspended in column buffer [20 mM Tris-HCl, 200 mM NaCl, 1 mM EDTA, 1 mM DTT, 1 mg/ml lysozyme, 1x Complete protease inhibitor cocktail (Roche)] and incubated on ice for 30-60 min before sonification lysis, followed by centrifugation for 30 min at 16,000 ***g*** to remove cell debris. The protein was bound to amylose resin (New England BioLabs) by incubation at 4°C for at least 1 h. The amylose beads were washed three times for 10-15 min with column buffer and the protein was eluted with 10 mM maltose in column buffer. Control MBP was prepared using the pMAL-c2x plasmid in parallel.

The Gαo[G203T] mutation was introduced by site mutagenesis using pQE32-Gαo (Kopein and Katanaev, 2009) as the template. The following primers were used: sense, AATTGTTTGACGTGACCGGTCAGCGCTC; antisense, GAGCGCTGACCGGTCACGTCAAACAATT. Recombinant *Drosophila* His_6-Gαo was prepared and preloaded with 1 mM GDP or GTPγS as described (Kopein and Katanaev, 2009); His_6-Gαo[G203T] was purified in parallel.

Pull-down assays

Thirty micrograms of His_6-Gαo were incubated with a twofold molar excess of MBP or MBP-Ank2_12 in HKB* buffer (100 mM KCl, 50 mM HEPES-KOH, 10 mM NaCl, 5 mM $MgCl_2$, 2 mM EGTA, 1 mM DTT, 5% glycerol, 0.5% NP40, 0.1% Tween) at 17°C for 1.5 h, prior to

addition to 100 µl 50% amylose resin slurry (New England BioLabs) pre-equilibrated with HKB* for an additional 1.5 h incubation at 17°C. The resin was washed four times with 1.5 ml HKB* for 15 min. The retained proteins were eluted by a 15 min incubation with 50 µl 10 mM maltose in HKB*. The proteins were resolved on 10% SDS-PAGE, electrotransferred to nitrocellulose membranes (Whatman) and detected by immunoblotting using rabbit anti-Gαo/i at 1:1000 (Merck). Equal loading was ensured by immunoblotting using rabbit anti-MBP antibodies at 1:4000 (New England Biolabs). For experiments with Gβγ, His6-Gαo was pre-incubated with the βγ dimer purified from porcine brains (Koval et al., 2010) for 45 min at room temperature before addition of equimolar amounts of MBP-Ank2_12 or MBP and incubation for an additional 1.5 h at 4°C. The pre-equilibrated resin was added to the proteins, incubated for 1.5 h and washed as described above. Proteins were eluted by addition of 5x sample buffer and boiling.

GTP-binding assay

Poorly hydrolysable fluorescent GTP analog Eu-GTP (PerkinElmer) was used in the GTP-binding assay with purified His_6-tagged *Drosophila* Gαo and Gαo[G203T] as described (Koval et al., 2010). The indicated (Fig. S2C) concentrations of the proteins were incubated for 2 hours in the presence of 5 nM GTP-Eu in 1xHKB buffer (10 mM HEPES-NaOH, 135 mM KCl, 10 mM NaCl, 2 mM EGTA, pH 7.5) supplemented with 5 mM $MgCl_2$. The reaction mixtures were subsequently transferred to AcroWell BioTrace NT 96-well plates (Pall), filtered using a vacuum manifold, and the membranes were washed twice with ice-cold washing buffer (20 mM Tris-HCl, 0.1 mM $MgCl_2$, pH 8.0). Fluorescence of the label retained on the membranes was measured immediately in a Victor^3 multilabel counter (PerkinElmer) in TRF mode. All experimental points were measured in duplicate. Curve fitting was performed in Prism 5 software (GraphPad).

Cell culture

Mouse neuroblastoma N2a cells were cultured in MEM supplemented with 10% FCS, L-glutamine and penicillin/streptomycin (all from Gibco, Life Technologies). Vector transfections were carried out with X-tremeGENE 9 (Roche) according to the manufacturer's instructions. Permanent AnkB or AnkG depletion in N2a cells was obtained by shRNA interference using annealed primers inserted into the *Bam*HI and *Hin*dIII sites of the pRetroSuper vector. Specific target sequences for murine *AnkB* and *AnkG* were previously described (Ayalon et al., 2008). Primers used for *AnkB*: 5′ sense strand, 5′-gatccccGAGTGGCCAACATCATATAttcaagagaTATATGATGTTGGCCACTCtttttta-3′; and 3′ antisense strand, 5′-agcttaaaaaGAGTGGCCAACATCATATAtctcttgaaTATATGATGTTGGCCACTCggg-3′. For *AnkG*: 5′ sense strand, 5′-gatccccGGCAGACAGACGCCAGAGCttcaagagaGCTCTGGCGTCTGTCTGCCtttttta-3′; and 3′ antisense strand, 5′-agcttaaaaaGGCAGACAGACGCCAGAGCtctcttgaaGCTCTGGCGTCTGTCTGCCggg-3′. A shluc vector expressing an shRNA against firefly luciferase was used as control. To generate stable lines, shRNA vectors were transfected into N2a cells and selection was performed in normal medium supplemented with 10 μg/ml puromycin. Downregulation of *AnkB* and *AnkG* expression was confirmed by standard RT-PCR methods using primers that recognize all ankyrin isoforms: ankB-For 5′-ACAGGTGATGGGGGAGAATAC-3′; ankB-Rev 5′-GAGTCCATTGTGTCTGCATCC-3′; ankG-For 5′-GCCTGCTCATAGGAAGAGGAA-3′; ankG-Rev 5′-GTCATGACCTTGTTGCAGAGC-3′. Primers to detect expression of the ribosomal protein S12 gene were used as control: S12-For 5′-GGGGCTAGCGCCACCATGGCCGAGGAAGGCATTGC-3′; S12-Rev 5′-GGGAGATCTTCATTTCTTGCATTTGAAATAC-3′.

Neurite outgrowth assay

N2a cells were co-transfected for 24 h with pEGFP-C1 (Clontech) and pcDNA3.1+ (Invitrogen) or a plasmid encoding human Gαo (Missouri S&T cDNA Resource Center). Cells were trypsinized and seeded on poly-L-lysine-coated coverslips for an additional 24 h to allow neurite formation. Alternatively, cells were transfected with EGFP-tagged ankyrin-B or ankyrin-G vectors (Ayalon et al., 2008) or co-transfected with the Gαo plasmid and prepared as above. Transient ankyrin double knockdowns were obtained by co-transfection of the shRNA-stably transfected N2a cell lines with the shRNA vectors shluc, shankB or shankG in addition to the plasmids described above. Ankyrin-independent neurite outgrowth was analyzed using an EGFP-tagged MARK2 (PAR1b) plasmid (Nishimura et al., 2012) under the transient double ankyrin knockdown conditions described above. Additionally, an mRFP-tagged MARK2 plasmid was generated by subcloning the *Bgl*II-*Kpn*I fragment including the MARK2 sequence into the same sites of the pmRFP-C1 vector. Then, N2a cells were co-transfected with the mRFP-MARK2 and AnkB-GFP plasmids and prepared as above. AnkB-GFP mean fluorescence intensities at the rear end of neurites (10-20 μm^2) and at whole neurites were scored from 40-80 neurites per condition using ImageJ, and the ratio values were used to determine AnkB-GFP accumulation at neurite tips in cells co-transfected with control pcDNA3.1+, Gαo or mRFP-MARK2 plasmids. For Nocodazole treatment, transfected N2a cells were allowed to adhere on coverslips for 6 h before incubation for an additional 18 h with Nocodazole (Sigma-Aldrich) in normal medium at the concentrations indicated in the corresponding figures. Cells were finally fixed with paraformaldehyde, stained with phalloidin-Rhodamine (Molecular Probes, Life Technologies) and DAPI (Sigma-Aldrich) or anti-Gαo antibody and mounted for microscopy analysis. Samples were recorded with an α-Plan-Apochromat 63x/1.4 or a Plan-Neofluar 20x/0.50 objective on an

AxioImager M1 microscope equipped with an AxioCam HRc camera and analyzed using AxioVision software (all from Zeiss). The number of transfected cells displaying neurites and lamellopodia, neurites per cell, total neurite length, and cell morphology were scored from 15-20 randomly taken images (>200 cells per condition).

Supplementary references

Ayalon, G., Davis, J. Q., Scotland, P. B. and Bennett, V. (2008) 'An ankyrin-based mechanism for functional organization of dystrophin and dystroglycan', *Cell* 135(7): 1189-200.

Brent, J. R., Werner, K. M. and McCabe, B. D. (2009) 'Drosophila larval NMJ dissection', *J Vis Exp*(24).

Chen, C. M., Strapps, W., Tomlinson, A. and Struhl, G. (2004) 'Evidence that the cysteine-rich domain of Drosophila Frizzled family receptors is dispensable for transducing Wingless', *Proceedings of the National Academy of Sciences of the United States of America* 101(45): 15961-6.

Dietzl, G., Chen, D., Schnorrer, F., Su, K. C., Barinova, Y., Fellner, M., Gasser, B., Kinsey, K., Oppel, S., Scheiblauer, S. et al. (2007) 'A genome-wide transgenic RNAi library for conditional gene inactivation in Drosophila', *Nature* 448(7150): 151-6.

Formstecher, E., Aresta, S., Collura, V., Hamburger, A., Meil, A., Trehin, A., Reverdy, C., Betin, V., Maire, S., Brun, C. et al. (2005) 'Protein interaction mapping: a Drosophila case study', *Genome Research* 15(3): 376-84.

Fremion, F., Astier, M., Zaffran, S., Guillen, A., Homburger, V. and Semeriva, M. (1999) 'The heterotrimeric protein Go is required for the formation of heart epithelium in Drosophila', *Journal of Cell Biology* 145(5): 1063-76.

Hornstein, N. J., Pulver, S. R. and Griffith, L. C. (2009) 'Channelrhodopsin2 mediated stimulation of synaptic potentials at Drosophila neuromuscular junctions', *J Vis Exp*(25).

Hummel, T., Krukkert, K., Roos, J., Davis, G. and Klambt, C. (2000) 'Drosophila Futsch/22C10 is a MAP1B-like protein required for dendritic and axonal development', *Neuron* 26(2): 357-70.

Katanaev, V. L., Ponzielli, R., Semeriva, M. and Tomlinson, A. (2005) 'Trimeric G protein-dependent frizzled signaling in Drosophila', *Cell* 120(1): 111-22.

Katanaev, V. L. and Tomlinson, A. (2006) 'Dual roles for the trimeric G protein Go in asymmetric cell division in Drosophila', *Proceedings of the National Academy of Sciences of the United States of America* 103(17): 6524-9.

Koch, I., Schwarz, H., Beuchle, D., Goellner, B., Langegger, M. and Aberle, H. (2008) 'Drosophila ankyrin 2 is required for synaptic stability', *Neuron* 58(2): 210-22.

Kopein, D. and Katanaev, V. L. (2009) 'Drosophila GoLoco-protein pins is a target of Galpha(o)-mediated G protein-coupled receptor signaling', *Molecular Biology of the Cell* 20(17): 3865-77.

Koval, A., Kopein, D., Purvanov, V. and Katanaev, V. L. (2010) 'Europium-labeled GTP as a general nonradioactive substitute for [(35)S]GTPgammaS in high-throughput G protein studies', *Analytical Biochemistry* 397(2): 202-207.

Lecuit, T., Brook, W. J., Ng, M., Calleja, M., Sun, H. and Cohen, S. M. (1996) 'Two distinct mechanisms for long-range patterning by Decapentaplegic in the Drosophila wing', *Nature* 381(6581): 387-93.

Mahr, A. and Aberle, H. (2006) 'The expression pattern of the Drosophila vesicular glutamate transporter: a marker protein for motoneurons and glutamatergic centers in the brain', *Gene Expr Patterns* 6(3): 299-309.
Mathew, D., Ataman, B., Chen, J., Zhang, Y., Cumberledge, S. and Budnik, V. (2005) 'Wingless signaling at synapses is through cleavage and nuclear import of receptor DFrizzled2', *Science* 310(5752): 1344-7.
Nishimura, Y., Applegate, K., Davidson, M. W., Danuser, G. and Waterman, C. M. (2012) 'Automated screening of microtubule growth dynamics identifies MARK2 as a regulator of leading edge microtubules downstream of Rac1 in migrating cells', *PLoS One* 7(7): e41413.
Packard, M., Koo, E. S., Gorczyca, M., Sharpe, J., Cumberledge, S. and Budnik, V. (2002) 'The Drosophila Wnt, wingless, provides an essential signal for pre- and postsynaptic differentiation', *Cell* 111(3): 319-30.
Pfeiffer, S., Ricardo, S., Manneville, J. B., Alexandre, C. and Vincent, J. P. (2002) 'Producing cells retain and recycle Wingless in Drosophila embryos', *Current Biology* 12(11): 957-62.
Pielage, J., Cheng, L., Fetter, R. D., Carlton, P. M., Sedat, J. W. and Davis, G. W. (2008) 'A presynaptic giant ankyrin stabilizes the NMJ through regulation of presynaptic microtubules and transsynaptic cell adhesion', *Neuron* 58(2): 195-209.
Pulver, S. R., Hornstein, N. J., Land, B. L. and Johnson, B. R. (2011) 'Optogenetics in the teaching laboratory: using channelrhodopsin-2 to study the neural basis of behavior and synaptic physiology in Drosophila', *Advances in physiology education* 35(1): 82-91.
Reichsman, F., Smith, L. and Cumberledge, S. (1996) 'Glycosaminoglycans can modulate extracellular localization of the wingless protein and promote signal transduction', *Journal of Cell Biology* 135(3): 819-27.
Schaefer, M., Petronczki, M., Dorner, D., Forte, M. and Knoblich, J. A. (2001) 'Heterotrimeric G proteins direct two modes of asymmetric cell division in the Drosophila nervous system', *Cell* 107(2): 183-94.
Schroll, C., Riemensperger, T., Bucher, D., Ehmer, J., Voller, T., Erbguth, K., Gerber, B., Hendel, T., Nagel, G., Buchner, E. et al. (2006) 'Light-induced activation of distinct modulatory neurons triggers appetitive or aversive learning in Drosophila larvae', *Current Biology* 16(17): 1741-7.
Strapps, W. R. and Tomlinson, A. (2001) 'Transducing properties of Drosophila Frizzled proteins', *Development* 128(23): 4829-35.
Zhang, B. and Stewart, B. (2010) 'Electrophysiological recording from Drosophila larval body-wall muscles', *Cold Spring Harb Protoc* 2010(9): pdb prot5487.
Zito, K., Parnas, D., Fetter, R. D., Isacoff, E. Y. and Goodman, C. S. (1999) 'Watching a synapse grow: noninvasive confocal imaging of synaptic growth in Drosophila', *Neuron* 22(4): 719-29.

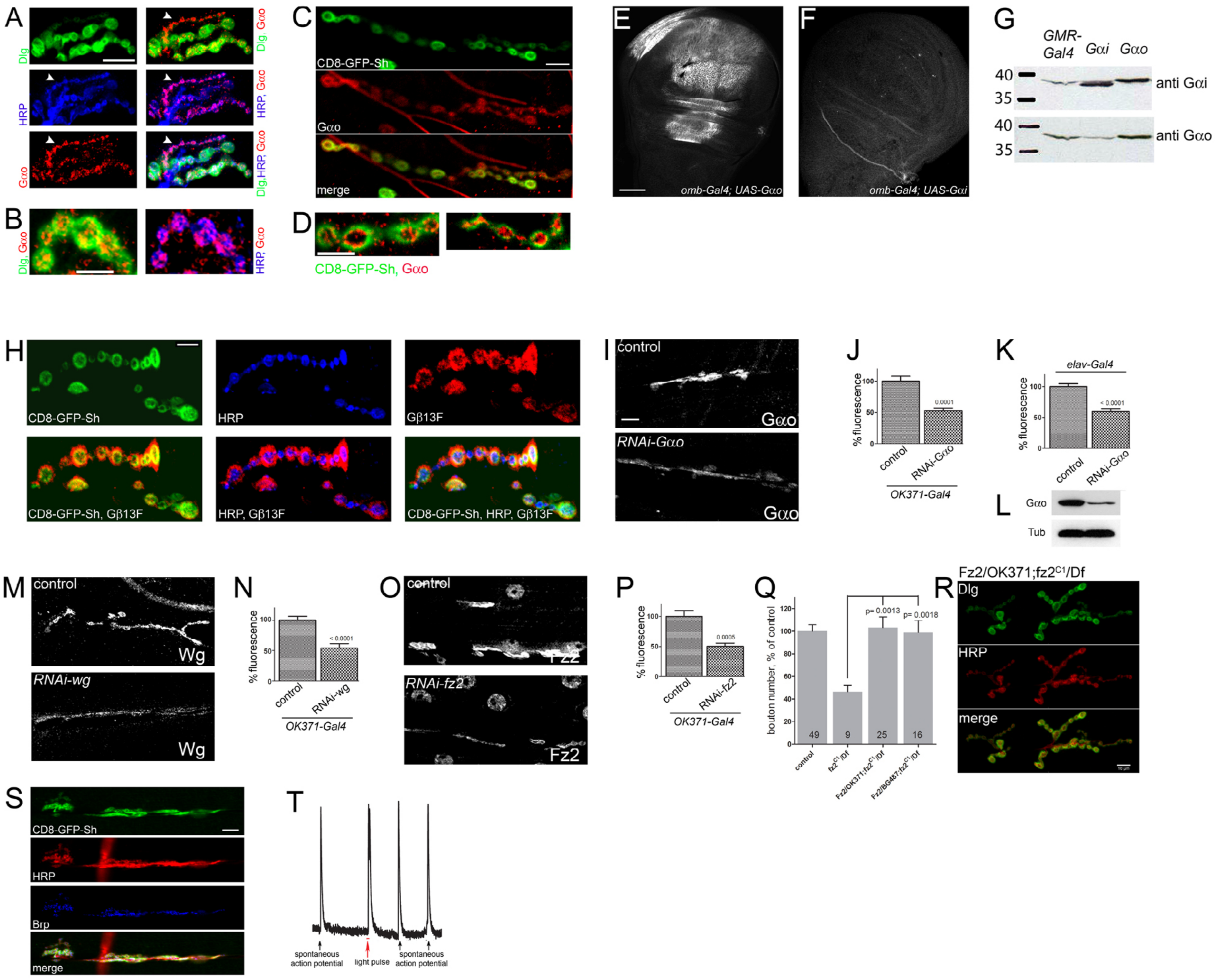
A
B
C
D
E
F
G
H
I
J
K
L
M
N
O
P
Q
R
S
T
CD8-GFP-Sh
merge
CD8-GFP-Sh, Gαo
omb-Gal4; UAS-Gαo
omb-Gal4; UAS-Gαi
GMR-Gal4
anti Gαi
anti Gαo
HRP
Gβ13F
CD8-GFP-Sh, Gβ13F
HRP, Gβ13F
CD8-GFP-Sh, HRP, Gβ13F
control
RNAi-Gαo
OK371-Gal4
elav-Gal4
% fluorescence
RNAi-wg
Wg
RNAi-fz2
Fz2
bouton number, % of control
Fz2/OK371;fz2C1/Df
Dlg
Brp
spontaneous action potential
light pulse

Figure S1. Characterization of the anti-Gαo antibodies and the RNAi lines.

(A) Gαo (red) is expressed in the presynaptic side of NMJ and is barely detected postsynaptically, as judged by colocalization with anti-HRP (blue) but only partial overlap with Dlg (green). Arrowhead points to the types Is boutons depicting low anti-Dlg staining. Scale bar 10µm. (B) High-magnification anti-Dlg (green), -Gαo (red), and -HRP (blue) staining. Scale bar 5µm. (C) Rabbit polyclonal antiserum raised against recombinant *Drosophila* Gαo produces the same staining pattern as shown in Figure 1. Scale bar 10µm. (D) High-magnification CD8-GFP-Sh (green) and anti-Gαo (red) staining. Scale bar 5µm. (E, F) The *omb-Gal4* driver was used to overexpress *Drosophila* Gαo (E) or Gαi (F) in wing imaginal discs. The anti-Gαo antibodies (Merck, raised against the C-terminal peptide corresponding to human Gαo, used in Figure 1) recognized overexpressed Gαo in the characteristic *omb* domain. In contrast, Gαi was not detected even with enhanced microscope settings. Scale bar 50µm. (G) Western blot of fly head extracts (wild type or overexpressing Gαo or Gαi) probed with antibodies against Gαo or Gαi. The anti-Gαo antibodies (Merck) recognize only the higher-migrating Gαo, both endogenous and overexpressed. The anti-Gαi antibodies (Merck) recognize both *Drosophila* Gαo and Gαi; Gαi migrates lower than Gαo and is not detectable in the non-Gαi-overexpressing samples. (H) Gβ13F (red) is found both in the presynapse colocalizing with anti-HRP (blue) and in the postsynapse colocalizing with, and even expanding the domain of, CD8-GFP-Sh (green). Scale bar 5µm. (I) Immunostainings of the wild-type (control) and *RNAi-Gαo*-expressing NMJ driven with *OK371-Gal4* at muscle 6/7 performed in parallel and recorded with identical microscope settings show a clear although incomplete downregulation in the levels of the proteins. (J) Quantification of the mean fluorescence in the NMJ indicates a two-fold decrease of fluorescence levels. Scale bar 10µm. (K) Expression of *RNAi-Gαo* with the driver *elav-Gal4* leads to a comparable decrease of Gαo in the NMJ determined by quantification of mean fluorescence. Western blot of fly head extracts expressing *RNAi-Gαo* with *elav-Gal4* however clearly demonstrates a drastic decrease in protein levels (L). (M-P) *RNAi-wg* and *RNAi-fz2* driven by *OK371-Gal4* also result in a clear downregulation of the Wg (M, N) and Fz2 (O, P) proteins as judged by immunostainings and quantification of the fluorescence in the NMJ. Immunostaining and image acquisition was performed like in (I, J). Anti-Fz2 antibodies have immunoreactivity in the muscle nuclei; note similar nuclear postsynaptic signal in control and *RNAi-fz2* samples (O). (Q) Quantification of total number of boutons show a rescue of the *fz2* mutant phenotype both by presynaptic and postsynaptic overexpression. (R) Representative image of presynaptic Fz2 rescue. Scale bar 10 µm. (S) Expression of Ptx in motoneurons results in reduced bouton numbers and aberrant NMJ morphology, similar to the phenotype of loss of Gαo (see Figure 2C). Scale bar 10µm. (T) Examples of spontaneous action potentials (black arrows) generated in the same recording session as a light-induced action potential (red arrow). The EJP amplitude and duration of the light-induced and spontaneous action potentials are similar. Scale as on Figure1G.

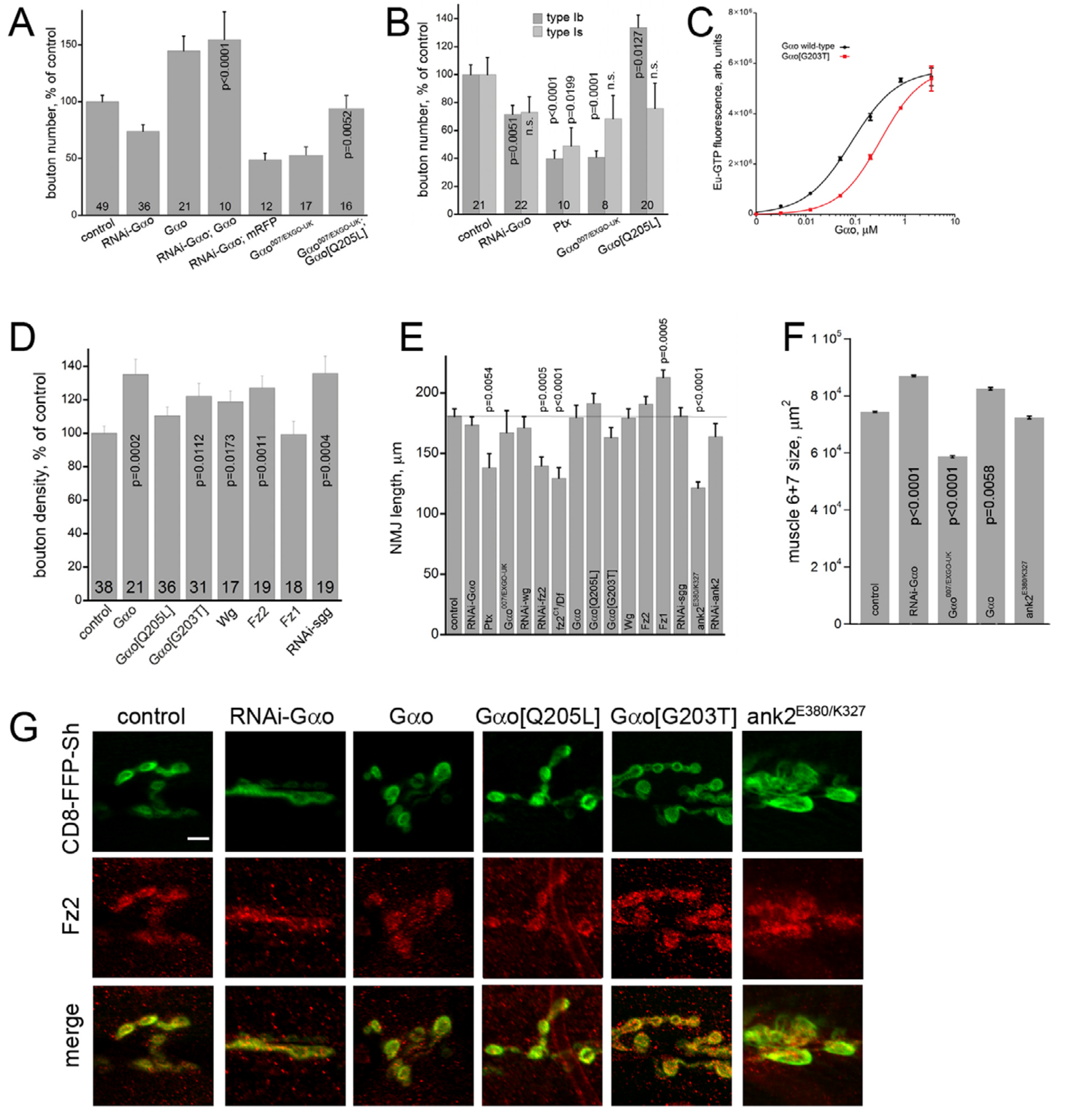

Figure S2. NMJ characterization and Fz2 localization in different genotypes; characterization of Gαo[G203T].

(A) The decrease of bouton number induced by downregulation of Gαo by RNAi is rescued by presynaptic co-overexpression of *Gαo* (p-value refers to the difference from *RNAi-Gαo*) to the level of *Gαo* overexpression. mRFP fails to restore bouton number of *RNAi-Gαo*. Pre-synaptic expression of activated Gαo rescues the *Gαo* genetic mutants (p-value refers to the difference from *Gαo*$^{-/-}$). (B) Change in type Ib and type Is bouton numbers upon alteration of Gαo levels or functionality. (C) Saturation binding curves of recombinant Gαo (black circles) and Gαo[G203T] (red squares) demonstrate that both proteins can be charged with Eu-GTP. Their affinities to the nucleotide analog differ, with that of the wild-type protein being about 4 times higher as judged by the calculated EC_{50} values of these proteins (83±8 nM for Gαo *vs* 316±37 nM for Gαo[G203T]). (D) Bouton density upon overactivation of the Wg pathway; p-values compared to the control are indicated. (E) Length of the NMJ of the indicated genotypes. (F) Muscle area of the indicated genotypes. p-values show significant differences to the control. (G) Localization of Fz2 in boutons of the denoted genotypes. Synaptic Fz2 localization is unaffected upon perturbations in Gαo or Ank2 levels. Scale bar 5μm.

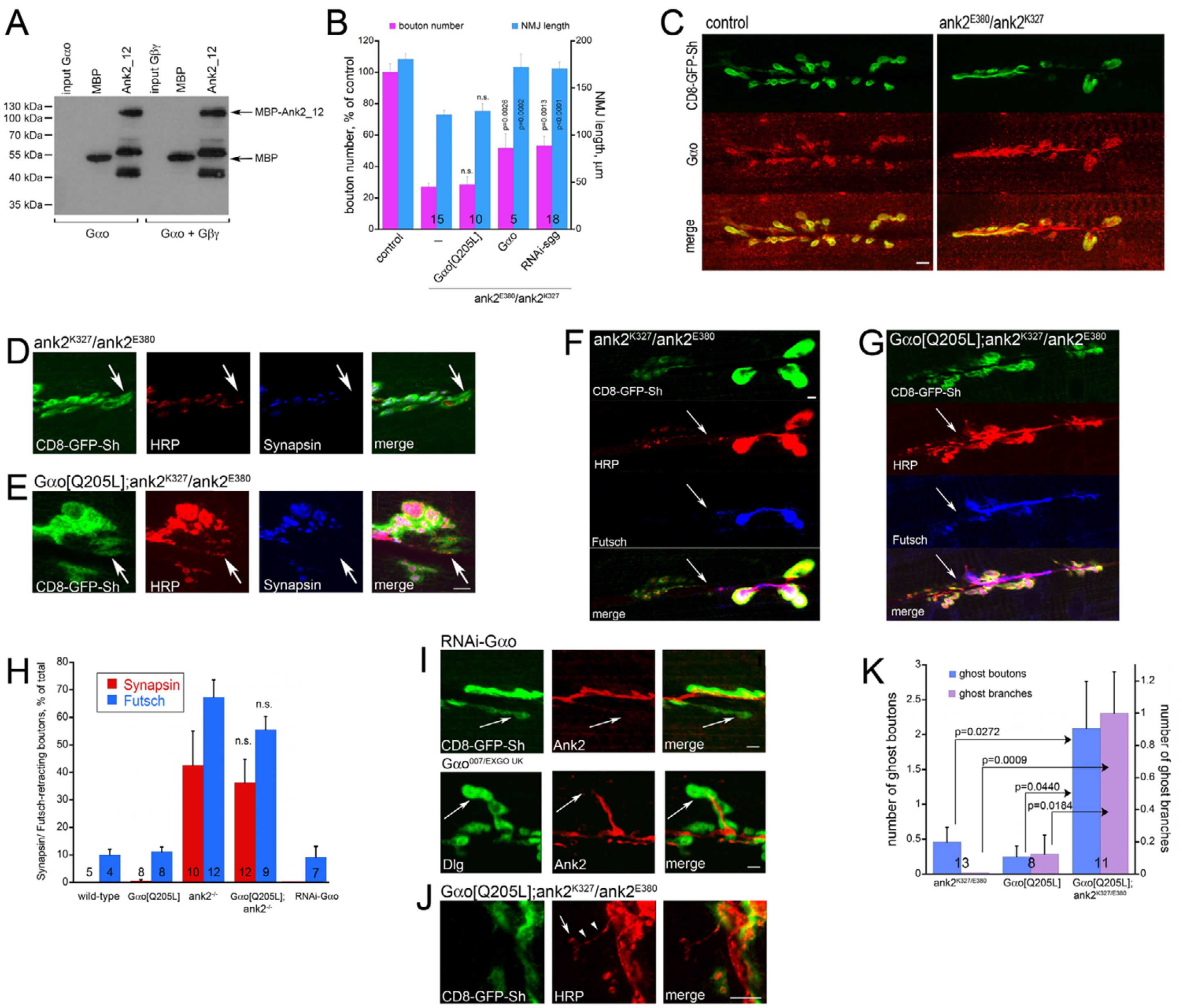

Figure S3. Additional controls to Western blotting, bouton quantification and analysis of presynaptic defects.

(A) Probing the western blot of Figure 5C with anti-MBP antibody demonstrates equal loading of the MBP control and MBP-Ank2_12. MBP-Ank2_12 is purified as a mixture of the full-length protein (ca. 120kDa) and two major degradation products. (B) Bouton number and NMJ length are increased in the *Gαo; ank2*$^{E380/K327}$ and *RNAi-sgg; ank2*$^{E380/K327}$ genotypes compared to *ank2*$^{E380/K327}$. Numbers of NMJ analyzed and p-values are indicated, n.s. means 'not significant'. (C) Synaptic Gαo localization is unaffected upon loss of Ank2. Scale bar 10 μm. (D-G) Synaptic retractions in *ank2*$^{-/-}$ (D, F) or *ank2*$^{-/-}$; *Gαo[Q205L]* (E, G) seen at the level of synaptic loss of Synapsin (D, E) or Futsch (F, G). Arrows in (D, E) point to some boutons having HRP but no Synapsin staining. Arrows in (F, G) point to the "border" where Futsch staining starts to be lost. Scale bar 5 μm. (H) Quantitation of synaptic retractions in different genotypes. (I) Ghost boutons (arrow) in boutons that still present in RNAi-Gαo and Gαo mutant NMJs. Scale bar 5 μm. (J) Neuronal processes (arrowheads) containing presynaptic HRP but lacking the postsynaptic structures can be seen upon overactivation of Gαo in the absence of Ank2. Scale bar 5 μm. (K) represents quantification of the phenotypes.

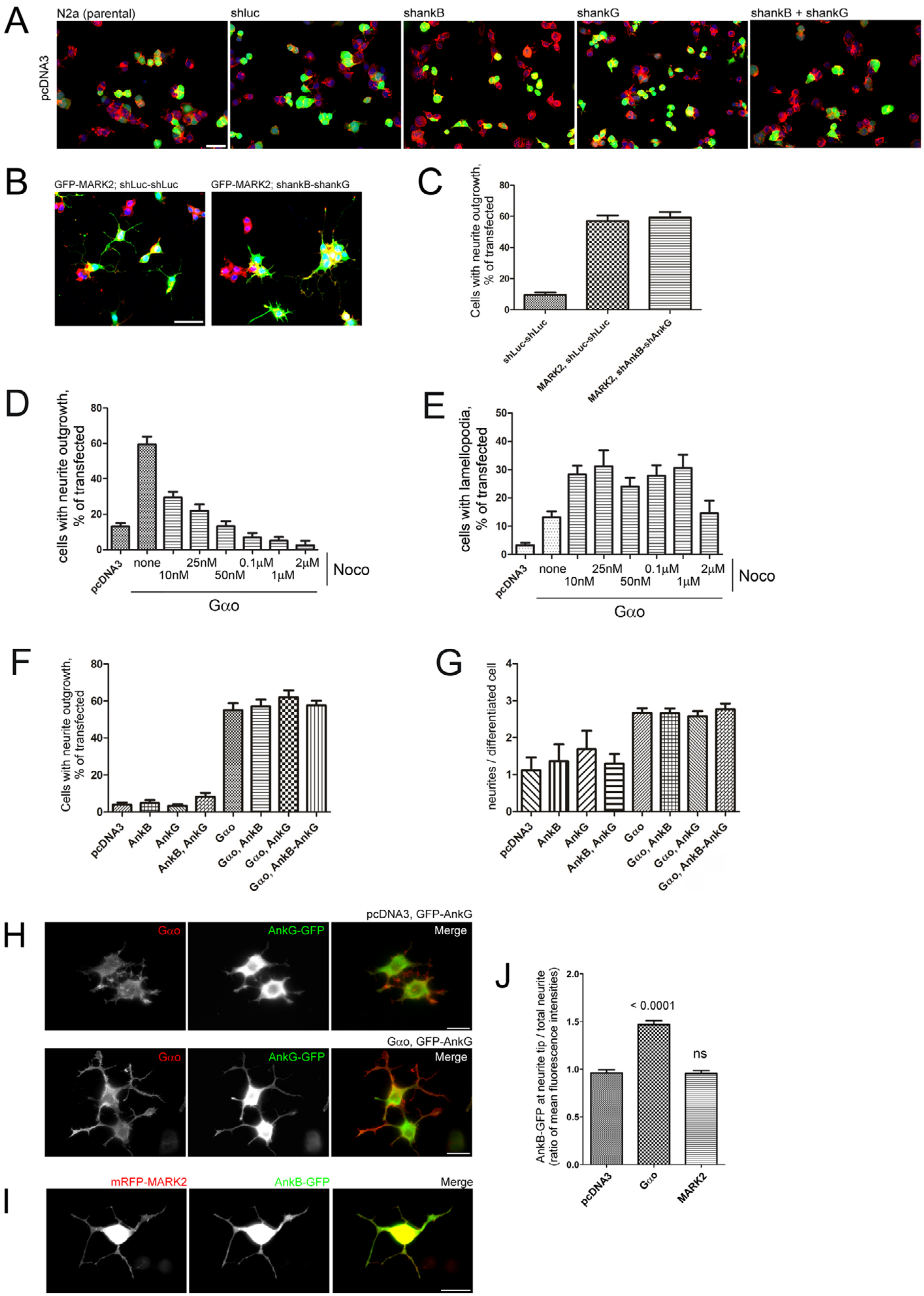
A
N2a (parental)
shluc
shankB
shankG
shankB + shankG
pcDNA3
B
GFP-MARK2; shLuc-shLuc
GFP-MARK2; shankB-shankG
C
Cells with neurite outgrowth, % of transfected
shLuc-shLuc
MARK2, shLuc-shLuc
MARK2, shAnkB-shAnkG
D
cells with neurite outgrowth, % of transfected
pcDNA3
none
10nM
25nM
50nM
0.1µM
1µM
2µM
Noco
Gαo
E
cells with lamellopodia, % of transfected
F
Cells with neurite outgrowth, % of transfected
AnkB
AnkG
AnkB, AnkG
Gαo, AnkB
Gαo, AnkG
Gαo, AnkB-AnkG
G
neurites / differentiated cell
H
pcDNA3, GFP-AnkG
AnkG-GFP
Merge
Gαo, GFP-AnkG
I
mRFP-MARK2
AnkB-GFP
J
AnkB-GFP at neurite tip / total neurite (ratio of mean fluorescence intensities)
< 0.0001
ns
MARK2

Figure S4. N2a cell treatments.

(A) Transfection of parental N2a cells with pcDNA3 empty vector poorly induces formation of neurites and lamellopodia. shRNA-stably transfected control (shluc) as well as *ankB* (shankB) and *ankG* (shankG) single and double knockdowns do not show any apparent phenotype after pcDNA3 transfection. Co-expression of EGFP (green) shows transfected cells and Rhodamine phalloidin (red) and DAPI (blue) are used to visualize F-actin and nuclei, respectively. Scale bar: 20 mm. (B) Overexpression of EGFP-tagged MARK2 (GFP-MARK2) induced neurite outgrowth in control shRNA-transfected (shluc-shluc) cells which is not affected in *ankB/G* double know-down (shankB-shankG) cells. Scale bar: 50 mm. (C) Quantification of the experiment described in (B). (D-E) Quantification of the Nocodazole (Noco) effects on neurite outgrowth (B) and lamellopodia formation (C) in Gαo overexpressing N2a cells. Nocodazole treatment blocks neurite outgrowth in a concentration dependent manner (B), while formation of lamellopodia is increased at any Nocodazole concentration (C). (F-G) Quantification of the effects on neurite outgrowth and neurite per cells by the overexpression of EGFP-tagged *ankB* (AnkB-GFP) or *ankG* (AnkG-GFP) in combination with Gαo or empty pcDNA3 vector. (H) Representative images of N2a cells overexpressing AnkG-GFP alone (pcDNA3, AnkG-GFP) or together with Gαo (Gαo, AnkG-GFP). Red fluorescence indicates Gαo immunostaining. (I) No accumulation of AnkB-GFP in neurite tips (and a more homogeneous distribution along the whole of the neurite instead) was observed upon overexpression of an mRFP-tagged MARK2 (mRFP-MARK2) construct (cf. Figure 7J). Scale bar: 10μm. (J) Ratio values of mean fluorescence intensities of AnkB-GFP at neurite tips vs. whole neurites indicate that AnkB significantly accumulates at the rear end of neurites formed by Gαo overexpression, but not at spontaneously formed neurites (pcDNA3) or at neurites induced by mRFP-MARK2 co-expression. P-value (Students t-test) is shown for Gαo co-overexpression. 'ns': non-significant ($P>0.05$).
